# Bayesian Variational Neural Architecture Search for Lightweight Weld Seam Feature Point Detection in Laser Vision Systems

**DOI:** 10.3390/s26196282

**Published:** 2026-10-03

**Authors:** Wenjia Mou, Yulin Wang, Chunyuan Liu, Yanbiao Zou

**Affiliations:** 1 Shien-Ming Wu School of Intelligent Engineering, South China University of Technology, Guangzhou 510640, China; 18934212075@163.com; 2 School of Mechanical & Automotive Engineering, South China University of Technology, Guangzhou 510640, China; cyliu2026@163.com (C.L.); ybzou@scut.edu.cn (Y.Z.); 3 School of Mechanical Engineering, Guangdong Ocean University, Zhanjiang 524088, China

**Keywords:** Bayesian variational inference, laser vision, lightweight model, neural architecture search, weld seam detection

## Abstract

In laser vision-based robotic welding, the seam feature point must be located, under strong arc-light and spatter interference, in every frame acquired while the weld is being made, so that the torch can be steered in real time; the detector must therefore keep pace with the camera on the modest processor of an industrial controller. Conventional deep learning models encounter structural redundancy and high computational latency under this constraint. To address these limitations, this paper proposes a lightweight object detection model, whose box-center output is mapped to the weld seam feature point, optimized via Bayesian variational neural architecture search (BayeNAS), in which architecture parameters are modeled as Gaussian distributions and updated through natural-gradient variational inference. A novel dual-selection optimization strategy is introduced to jointly search for optimal candidate operators and network topology branches, achieving an explicit balance between model complexity and hardware latency. Furthermore, an in-search channel pruning strategy is incorporated to compress the search space and prevent the combinatorial growth of the channel configuration space. Comparative analysis and real-world welding experiments demonstrate that the proposed model achieves a high processing speed of 78 frames per second (FPS) on a legacy central processing unit (CPU)—above the 60 fps acquisition rate of the sensor—while maintaining a mean positioning error within 0.15 mm on the measurement plane. These results demonstrate that the proposed model achieves detection accuracy comparable to heavyweight detectors while offering an order-of-magnitude advantage in inference speed and model size, validating its suitability for low-cost industrial deployment.

## 1. Introduction

**Figure 1:**
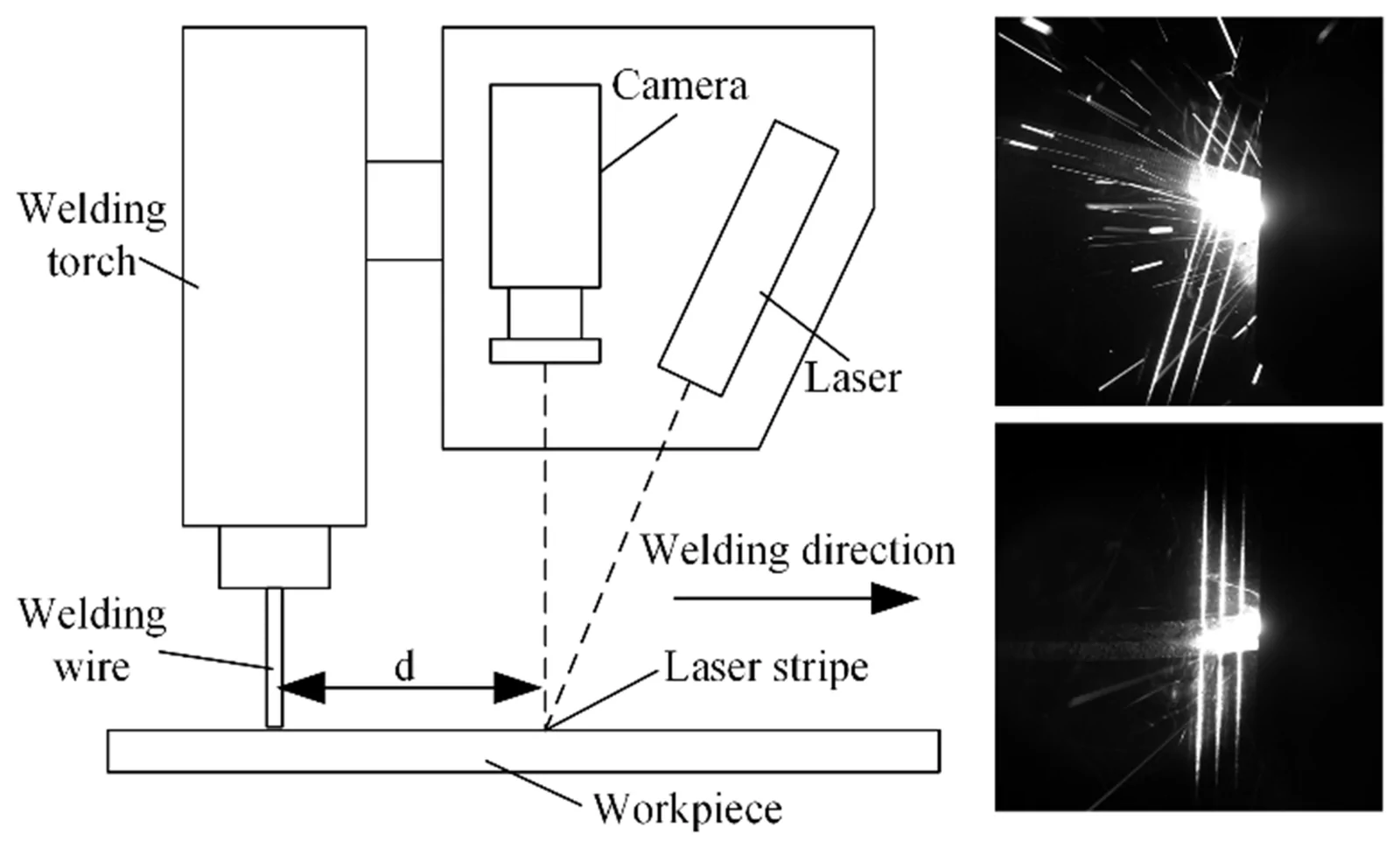
Overview of laser vision system–based weld seam tracking.

Welding technology is a fundamental component of modern manufacturing, playing a critical role in sectors such as shipbuilding, automotive manufacturing, and aerospace engineering [1]. Traditional manual welding methods are increasingly insufficient to meet the demands of modern industry due to low production efficiency and high labor intensity [2]. Recently, welding robot systems integrated with visual sensing technology have developed rapidly, enabling automated and efficient seam identification and tracking in complex environments. Among these, laser vision systems (as shown in Figure 1) have been widely adopted because they project structured light onto the workpiece, producing high-contrast laser stripe patterns, offering high measurement accuracy and strong noise robustness.

Despite these advancements, achieving high accuracy and real-time performance simultaneously on resource-constrained industrial platforms remains a significant challenge. This limitation was already identified in an earlier survey [2] and continues to be singled out in the most recent state-of-the-art reviews: on-line computational cost is listed alongside noise robustness and calibration as a principal obstacle to industrial deployment [3], the adaptability and real-time capability of vision algorithms are identified as open problems for autonomous arc welding [4], and the trade-off between model complexity and deployability on industrial controllers is highlighted as a key direction for automated robotic welding [5]. The steady stream of lightweight seam-detection models published in the past year alone [6,7] confirms that the problem has not been closed. Traditional digital image processing methods often rely on manually designed filters and trial-and-error parameter tuning, which lack the flexibility to handle severely noise-corrupted images. While deep learning models offer superior feature extraction capabilities, manually designed architectures often suffer from structural redundancy and high inference latency. Specifically, such models frequently adopt uniform channel configurations across all layers, ignoring the varying feature redundancy at different network depths. Recent lightweight detectors for weld seam sensing are still obtained by substituting a backbone and pruning channels by hand [6,8,9], or by pruning a fixed architecture after training [7], a procedure that must be repeated whenever the sensing configuration or the deployment platform changes (Section 2). The gap addressed in this work is therefore the absence of a procedure that constructs the detector automatically under an explicit constraint on the inference latency of the target device, rather than by manual redesign for each new configuration.

This work addresses in-process guidance rather than post-process inspection. The sensor measures the geometry of the joint ahead of the weld pool in order to steer the torch, and no claim is made regarding the internal quality of the resulting weld, which remains the province of radiographic or ultrasonic examination. The two are complementary: misalignment between torch and joint is a recognized contributor to a lack of fusion and incomplete penetration, so that accurate in-process tracking acts upstream of defects that surface examination cannot reveal.

To overcome these limitations, this study introduces an automatic model construction approach using Bayesian Variational Neural Architecture Search (BayeNAS). To balance accuracy and inference speed, a dual-selection optimization strategy is proposed for operation operators and network branches, explicitly incorporating inference latency on target deployment devices as a constraint. Additionally, a model channel pruning technique is integrated into the search process to mitigate the excessive search space and further compact the model.

The main contributions of this study are as follows: First, a Bayesian variational NAS framework (BayeNAS-Weld) is developed for weld seam feature point detection, which jointly learns posterior distributions over both candidate operators and topology branches—extending prior variational NAS methods [10] that search operators only. Second, a dual optimization strategy for operators and branches is proposed, with inference latency on target devices incorporated as a core constraint. Meanwhile, a joint channel pruning strategy is introduced to address the excessive search space, further enhancing computational efficiency. Third, a robotic weld seam tracking experimental platform is established, and practical welding experiments on butt and lap joints are conducted. The results are reported in Section 4.

## 2. Related Work

Weld seam recognition and feature extraction methods based on laser vision systems can be broadly classified into two categories: strategies relying on traditional digital image processing algorithms and intelligent methods based on deep learning models.

In traditional approaches, researchers manually design filter operators to extract features based on geometric characteristics and pixel intensity. For instance, Chen et al. [11] designed a convolution kernel operator to estimate weld seam likelihood, utilizing Non-Maximum Suppression (NMS) to preselect candidates. Li et al. [12] proposed the internal propulsion center extraction (IPCE) algorithm to rapidly extract laser stripes while reducing arc and spatter noise. Other scholars have employed slope analysis with the least squares method [13], Kalman filtering to identify feature stripes [14], and Laplacian of Gaussian (LoG) [15] filters combined with non-uniform rational B-spline (NURBS)-snake algorithms to improve detection accuracy. However, these methods typically require a tedious trial-and-error process for parameter initialization and depend heavily on designer experience. Such geometric strategies remain in active development. Chen et al. [16] combined a dynamic region of interest propagated from the preceding frame with a slope-constrained RANSAC fit, suppressing arc and spatter interference without any learned component, and Liu et al. [17] embedded a laser-vision tracking model within a global weld-planning strategy to follow complex curved seams with an accuracy of 0.4 mm. Furthermore, manually tailored operators often lack flexibility and fail to handle severely noise-corrupted images common in real-world welding as shown in Figure 1.

Deep learning enables end-to-end learning from raw data, automatically capturing complex features without extensive manual engineering. For multi-pass welding, Liu et al. [18] designed a restoration and extraction network (REN) based on conditional generative adversarial network (cGAN) principles to extract clean stripes from noisy images. Xiao et al. [19] developed the LSFP-tracker using a Siamese network to produce high-resolution feature maps for accurate tracking. Additionally, the laser stripe edge guidance network (LSEGN) model based on an improved VGG (Visual Geometry Group) net was proposed for autonomous recognition of multi-pass seams [20]. To enhance efficiency, Zou et al. [21] introduced a lightweight network based on SOLOv2 for segmentation, while Xiao et al. [22] adapted the classical Faster R-CNN for continuous and discontinuous seam detection. More recently, general-purpose keypoint and pose estimation architectures have been transferred directly to this task: Baek and Moon [23] applied a YOLOv8-pose model with a dedicated noise-filtering stage to shipbuilding seam tracking, reporting a mean absolute error of 0.128 mm, and Yang et al. [24] adapted a YOLO-based detector to multi-type seam keypoints in noisy welding scenes. Despite their success, conventional deep learning models are often manually designed, requiring significant time for architecture tuning. Applied to welding, these models often suffer from structural redundancy or insufficient feature extraction for specific scenarios.

This limitation has shaped the most recent literature, and it is recognized as an open problem in the current review literature. Guo et al. [3] list on-line computational cost, alongside noise robustness and calibration, among the principal obstacles to the industrial deployment of vision sensing in robotic welding. Ma et al. [4], in a state-of-the-art review of vision-based autonomous robotic arc welding, reach a comparable conclusion regarding the real-time capability of the perception stage, and Li et al. [5], surveying the key technologies of automated robotic welding, likewise identify the deployability of vision algorithms on industrial hardware as a continuing constraint. The prevailing response has been to compress otherwise accurate networks by hand. Chen et al. [25] designed DSNet, a hybrid convolutional–transformer architecture for real-time weld seam segmentation in which the transformer branch is deliberately narrowed so that the model retains real-time throughput. Zhao et al. [8] replaced the backbone of YOLOv8s-Seg with MobileNetV3, reconstructed the C2f module, manually pruned its output channels and re-introduced an attention block to recover the accuracy lost to compression, obtaining a 4.88 MB model that completes segmentation and path fitting in 54 ms on a Jetson Nano. Gao et al. [9] proposed WeldLight, a hand-designed one-stage lightweight network with a noise-filtering attention module for single-line structured-light images, reporting mean absolute positioning errors of 1.639 and 1.736 pixels on low- and high-noise test sets at 29.32 ms per frame on a CPU.

This design practice has persisted into the most recent work. Wang et al. [6] hand-designed a lightweight C3k2-based backbone together with a non-parametric peak-refinement decoder for seam keypoint detection, obtaining a 1.4 M-parameter model that runs at 17.01 ms per frame on a CPU with a mean absolute error of 1.89 pixels. Yang et al. [24] adapted a YOLO-based detector to multi-type seam keypoints in noisy welding scenes and report 159.80 FPS; this throughput, however, is obtained at 19.42 GFLOPs on a laptop GPU, so that the real-time behavior is contingent on hardware acceleration rather than on the compactness of the network itself. Chen and Zou [7] moved a step further toward automation by performing automated structured pruning within a co-optimization framework, reaching 55.2 FPS and a tracking error of 0.154 mm; there, however, the pruning is applied to a manually fixed segmentation architecture, so that the choice of operators and of the network topology is still made a priori. In each of these works the macro-architecture is the product of manual design: a backbone is substituted, channels are pruned by hand or, at best, pruned automatically within a topology that was itself fixed in advance, and an attention module is appended to compensate for the accuracy lost to compression. This is a trial-and-error cycle that must be repeated whenever the sensing configuration or the deployment hardware changes, and in which the compression decisions are taken a priori and applied uniformly across depths rather than being derived from the latency characteristics of the target device.

Automatic Neural Architecture Search (NAS) has emerged to explore optimal network structures within predefined search spaces [26]. Howard et al. [27] utilized reinforcement learning combined with manual refinements to obtain the lightweight MobileNetV3. This model features low memory consumption and high inference speed, making it suitable for deployment on lightweight platforms. However, reinforcement learning–based search algorithms require extensive computational resources during training, leading to high search costs. Liu et al. [28] defined a supernetwork and searched for optimal subnetworks using a differentiable approach. This method relaxes the discrete search space into a continuous one, allowing the supernetwork to optimize architecture parameters via Gradient Descent and significantly reducing training time. However, this approach prioritizes accuracy and does not account for the computational cost and inference latency of subnetworks. Wu et al. [29] incorporated real hardware latency into a differentiable NAS framework, jointly considering model accuracy and inference time to search for an optimal lightweight network for specific hardware. Hardware-aware search has since been extended to increasingly constrained platforms. King et al. [30] combine differentiable search with latency lookup tables and dynamic convolutions to generate architectures that satisfy user-specified limits on execution latency and peak memory on microcontrollers. Xiang et al. [31] likewise target a constrained instruction-set architecture, combining a latency lookup table with a learned latency predictor to search under an explicit latency budget without network training. The fidelity of such latency proxies has itself become a subject of study: Akhauri and Abdelfattah [32] show that latency predictors transfer poorly across devices and propose an end-to-end training strategy that improves prediction accuracy by 22.5% on average over twelve prediction tasks. The present work adopts the lookup-table formulation for its differentiability and low search cost, and states its limitations explicitly in Section 4.6. Although differentiable architecture search offers high training efficiency, it suffers from instability, and performance often collapses as the search progresses [33]. This collapse typically arises because deterministic gradient optimization tends to overfit specific operators (e.g., skip connections) and get trapped in local optima. To address this, Zhang et al. [10] proposed BaLeNAS, which reformulates differentiable architecture search as a distribution-learning problem solved via the Bayesian learning rule with natural-gradient variational inference. Unlike deterministic approaches, this method models architecture parameters as probability distributions rather than fixed values. This strategy naturally introduces randomness via sampling, effectively reducing the risk of convergence to suboptimal solutions and enhancing global optimization capability. Inspired by these developments, this study extends the Bayesian variational search paradigm with a latency-aware dual-selection mechanism (over both operators and branches) and integrated channel pruning to build a highly efficient and compact model for weld seam detection.

While the proposed framework builds upon several established techniques, its formulation differs from each of them in scope and objective. Differentiable architecture search (DARTS) [28] relaxes the discrete architecture space into a continuous one but optimizes deterministic architecture parameters, which is prone to performance collapse; BaLeNAS [10] addresses this by learning Gaussian distributions over operator parameters via the Bayesian learning rule, yet it considers operator selection only and is hardware-agnostic. FBNet [29] incorporates a lookup-table latency loss into differentiable NAS, but relies on deterministic Gumbel-softmax relaxation and searches operators within a fixed macro-topology. Network slimming [34] prunes channels via BN scaling factors, but only as a post-hoc compression step applied to a fixed architecture. The SSD detection head [35] is adopted here as a standard component and is not claimed as a contribution.

Bayesian methods are well established in welding research, although they have been applied to a different class of variables than those considered here. Haas et al. [36] use Bayesian optimization to select laser welding process parameters, reducing the number of experiments required to reach a specified weld depth. Cao et al. [37] estimate the state of GTAW and GMAW processes from weld pool images by Bayesian filtering, and Goumeidane et al. [38] discriminate defect classes in weld radiographs using Bayesian networks. In each case the posterior is placed over a physical quantity—a process parameter, a process state or a defect class—and is evaluated for each weld or each workpiece. The present work places the posterior over architecture variables instead and evaluates it once, at design time; its output is not an estimate of the process but a network that subsequently runs deterministically inside the tracking loop. Bayesian inference has thus been used in welding to optimize and to monitor the process, but not to construct the detector that closes the tracking loop, and in particular not under an explicit constraint on the inference latency of the controller that must execute it.

The contribution of this work is therefore not a new optimization principle, but a unified search framework tailored to laser-vision weld seam detection: (i) the variational treatment is extended from operator parameters α to an additional set of branch/topology parameters β, enabling joint operator-and-topology search within one posterior; (ii) a device-specific latency term is embedded directly into the variational objective, coupling the Bayesian search with hardware constraints; and (iii) BN-based channel pruning is performed inside the search loop rather than as post-processing, jointly shrinking the search space and the final model. To the best of the authors’ knowledge, the combination of these three mechanisms for constructing a CPU-deployable weld seam detector has not been reported. This remains the case in the most recent literature on lightweight seam detection [6,7,24], in which architectures are obtained by manual design or by pruning within a fixed topology rather than by search.

## 3. Methodology

The proposed BayeNAS-Weld framework aims to automatically construct a lightweight weld seam detection model by balancing positioning accuracy and inference efficiency. This section details the system integration, the design of the searchable supernetwork, and the Bayesian variational search strategy incorporating latency constraints and channel pruning.

### 3.1. System Setup and Image Acquisition Framework

**Figure 2:**
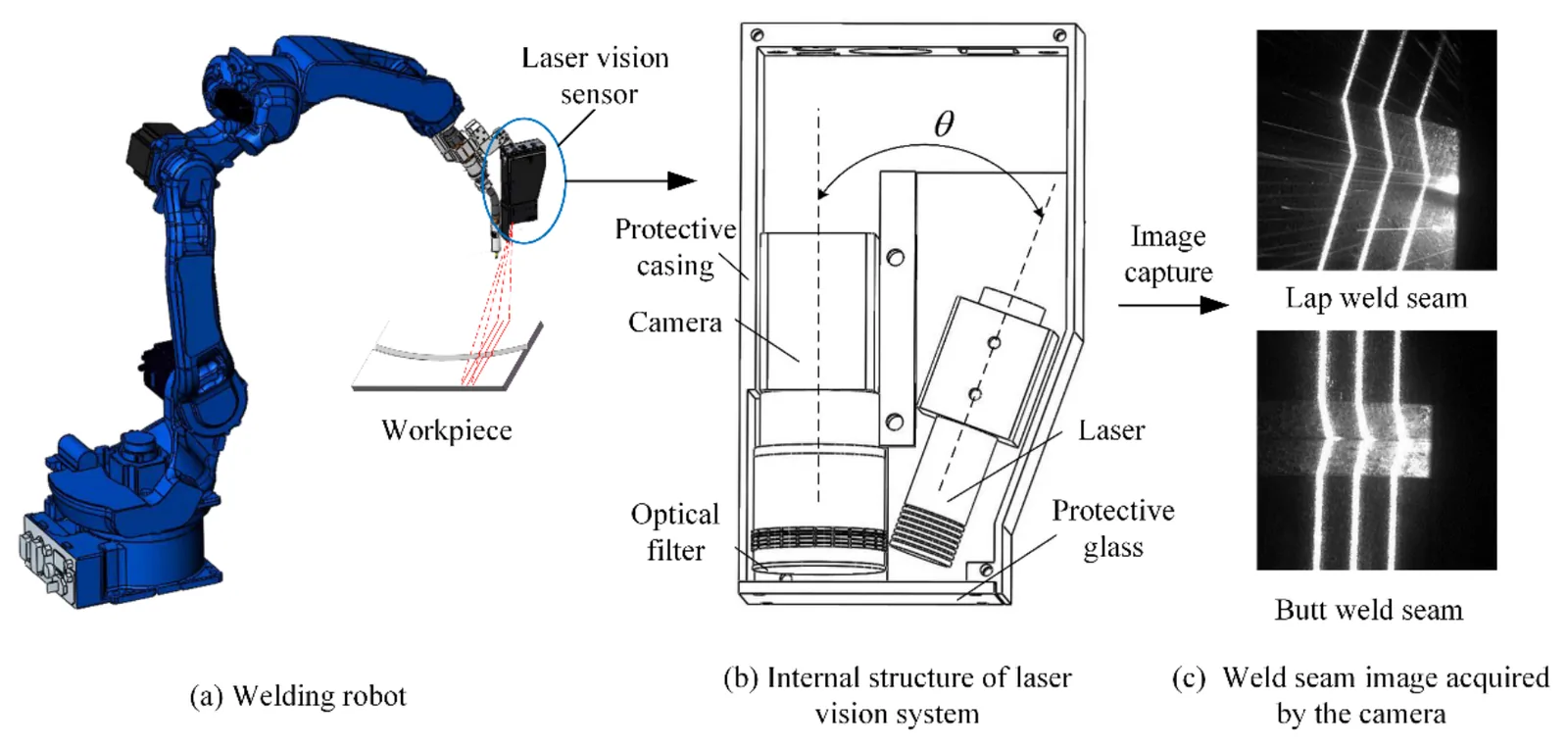
The experimental platform.

To achieve high-precision seam tracking, a closed-loop system integrating hardware sensing and algorithmic optimization was established. The experimental platform consists of a Yaskawa six-axis arc welding robot (MOTOMAN-MA1440) and a laser vision system mounted as the end effector, as shown in Figure 2. The laser vision sensor includes an industrial camera, a triple-line laser generator, an optical filter, and protective housing. During operation, the triple-line laser projects parallel beams onto the workpiece, encoding both two-dimensional position and depth information into laser stripe patterns. Table 1 lists the models and parameters of the main components of the laser vision sensor.

In the image acquisition process, the distance *d* between the laser stripes and the weld pool is set to 15 mm. This forerun distance is not a free parameter to be maximized: it is bounded below by the requirement that the laser stripes fall on unmelted parent material ahead of the weld pool and its immediate spatter field, and bounded above by the fact that the measurement point and the point at which the torch acts are separated by d. Reducing d increases the in-band radiant background at the stripe location, which for a compact arc and pool source scales approximately as 1/d^2^, and increases the rate at which spatter contaminates the protective glass. Increasing d, conversely, lengthens the interval between measurement and actuation—at the traverse speed of 15 mm/s used here, d = 15 mm corresponds to a lead time of 1.0 s—and makes the transferred correction more sensitive to seam curvature and to residual error in the sensor-to-torch transform; the mitigation of such sensor forerun is an explicit design objective in recent seam-tracking systems [39]. The value of 15 mm was therefore selected as the smallest forerun distance at which reliable stripe extraction had been maintained under strong arc light and spatter in previous work with this sensor configuration, where tracking errors of 0.5 mm and ±0.2 mm were reported at this distance [40,41]. The captured images are transmitted to an industrial computer for real-time processing. To reduce the computational load for the subsequent Neural Architecture Search (NAS), the raw images are cropped to a resolution of 192×192 pixels. This preprocessing step ensures that the input data distribution is consistent and optimized for the lightweight detection model.

Prior to deployment, the vision system was calibrated as follows. The camera intrinsics were estimated using Heikkilä’s method for circular control points [42] with a 36 mm planar circular-dot target. The laser planes of the triple-line generator were calibrated by plane fitting on the reconstructed point cloud. The transformation from the sensor frame to the robot base frame was obtained through the hand-eye calibration procedure of Zou and Chen [43], in which the rotation matrix is first estimated by ordinary least squares and its orthogonality is subsequently enforced through a semidefinite program (SDP); the average residual, defined as the Euclidean distance between the calibration points reconstructed by the sensor and their positions measured by the robot, was below 0.5 mm.

Once the images and the corresponding robot poses have been acquired, every stage of this procedure is solved automatically: the dot centres are detected and the intrinsics estimated without operator input, the laser planes are obtained by least-squares fitting, and the SDP formulation of the hand-eye problem is convex, so that a unique global optimum is returned without an initial guess or manual parameter tuning. Operator intervention is therefore confined to positioning the calibration target and commanding the robot through the acquisition poses. This acquisition stage can itself be automated: Xiao et al. [44] drive the acquisition of calibration images from the robot motion equation together with a motion-range constraint and refine the parameters by visual servoing, reporting a substantial improvement in calibration efficiency for a laser vision sensor in a robotic welding system. Such a scheme was not implemented here because the calibration is performed once, when the sensor is mounted, rather than per workpiece or per weld; integrating automatic acquisition is nevertheless a worthwhile step toward field deployment. The effect of the residual calibration error on the results reported below is analyzed in Section 4.5. At the working distance of 120 mm, the effective scale factor on the measurement plane is approximately 0.0754 mm/pixel; the 192×192 cropped region thus corresponds to a field of view of approximately 14.5×14.5 mm. This factor is used throughout the paper to convert image-space positioning errors (in pixels) into physical errors (in millimeters).

The sensor does not image the arc or the weld pool. The camera is directed at the laser stripes projected onto the unmelted parent material ahead of the pool, and it is the deformation of those stripes at the joint, not any thermal signature, that is measured. The laser vision sensor is not a commercial unit; it was developed in-house from the components listed in Table 1. Its resistance to arc light rests on three measures. Optically, the triple-line generator emits 100 mW at 660 nm and the camera carries a BNP660 bandpass filter whose pass band is centered on 660 nm with a bandwidth of 10 nm, so that the broadband radiation of the arc is rejected outside that band. Geometrically, the forerun distance of 15 mm places the imaged stripes ahead of the weld pool, so that the pool and the majority of the spatter fall outside the region of interest, while a polycarbonate protective glass and an aluminum-alloy housing shield the optics from spatter and radiated heat. Algorithmically, the dataset described in Section 4.1 was itself acquired under strong arc light and spatter, so that the searched model is trained on the noise conditions under which it operates.

### 3.2. Supernetwork Definition and Search Space

To construct an object detection model tailored for weld seam identification while mitigating the high costs associated with manual design, this study employs a supernetwork-based neural architecture search (NAS) [26]. Following the differentiable formulation introduced by Liu et al. [28], the discrete architectural choices are relaxed into continuous weights, so that the architecture can be optimized by gradient descent rather than by discrete search. A further advantage of this framework is parameter sharing, introduced by Pham et al. [45] and analyzed in the one-shot setting by Bender et al. [46]: candidate subnetworks share weights within a single supernetwork, which avoids the prohibitive computational expense of training multiple independent models from scratch. The search space defined in this subsection is deliberately conventional—the supernetwork and continuous relaxation follow DARTS [28], the detection paradigm follows SSD [35], and the candidate operator set follows established lightweight design [27]—so that the contribution of the search strategy introduced in Section 3.3, Section 3.4 and Section 3.5 can be assessed independently of it.

#### 3.2.1. Overall Supernetwork Architecture

**Figure 3:**
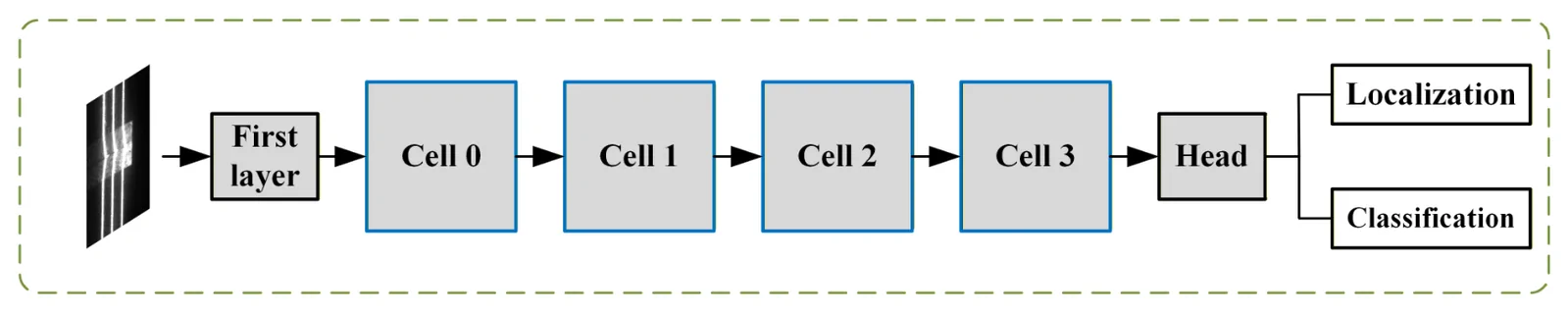
Overall supernetwork architecture.

The proposed detection framework is built upon the Single Shot Multibox Detector (SSD) architecture [35], adapted specifically for the geometric characteristics of laser-vision weld seam images. As illustrated in Figure 3, the supernetwork comprises six primary module layers arranged sequentially.

The “First layer” and the prediction “Head” are designed with fixed architectures to ensure the stability of input processing and output generation. Between these fixed endpoints lie four searchable modules, denoted as Cell 0 through Cell 3. These cells constitute the core search space where the network topology is automatically optimized to fit the data distribution. The detailed configuration of each network stage, including feature map resolutions, channel dimensions, and operator types, is presented in Table 2.

**Table 2:** Configuration of each network stage: search space, selected operators, and channel widths before and after pruning.

Stage	Output Size	Stride	Channels (Supernetwork)	Channels (Final Sub-Model)	Candidate Operators	Selected Operator
First layer	192×192	1	8, 16	8, 16	*Fixed*	Conv 3×3, Conv 1×1
Cell 0	96×96	2	32	16	Conv 3×3, Conv 5×5, Sep_Conv 3×3, Sep_Conv 5×5	Conv 3×3
Cell 1	48×48	2	64	39	Conv 3×3, Conv 5×5, Sep_Conv 3×3, Sep_Conv 5×5	Conv 5×5
Cell 2	24×24	2	128	72	Conv 3×3, Conv 5×5, Sep_Conv 3×3, Sep_Conv 5×5	Conv 5×5
Cell 3	12×12	2	128	66	Conv 3×3, Conv 5×5, Sep_Conv 3×3, Sep_Conv 5×5	Conv 5×5
Head	7×7	2, 1	128, 7	128, 7	*Fixed*	Conv 3×3, Conv 3×3

**Figure 4:**
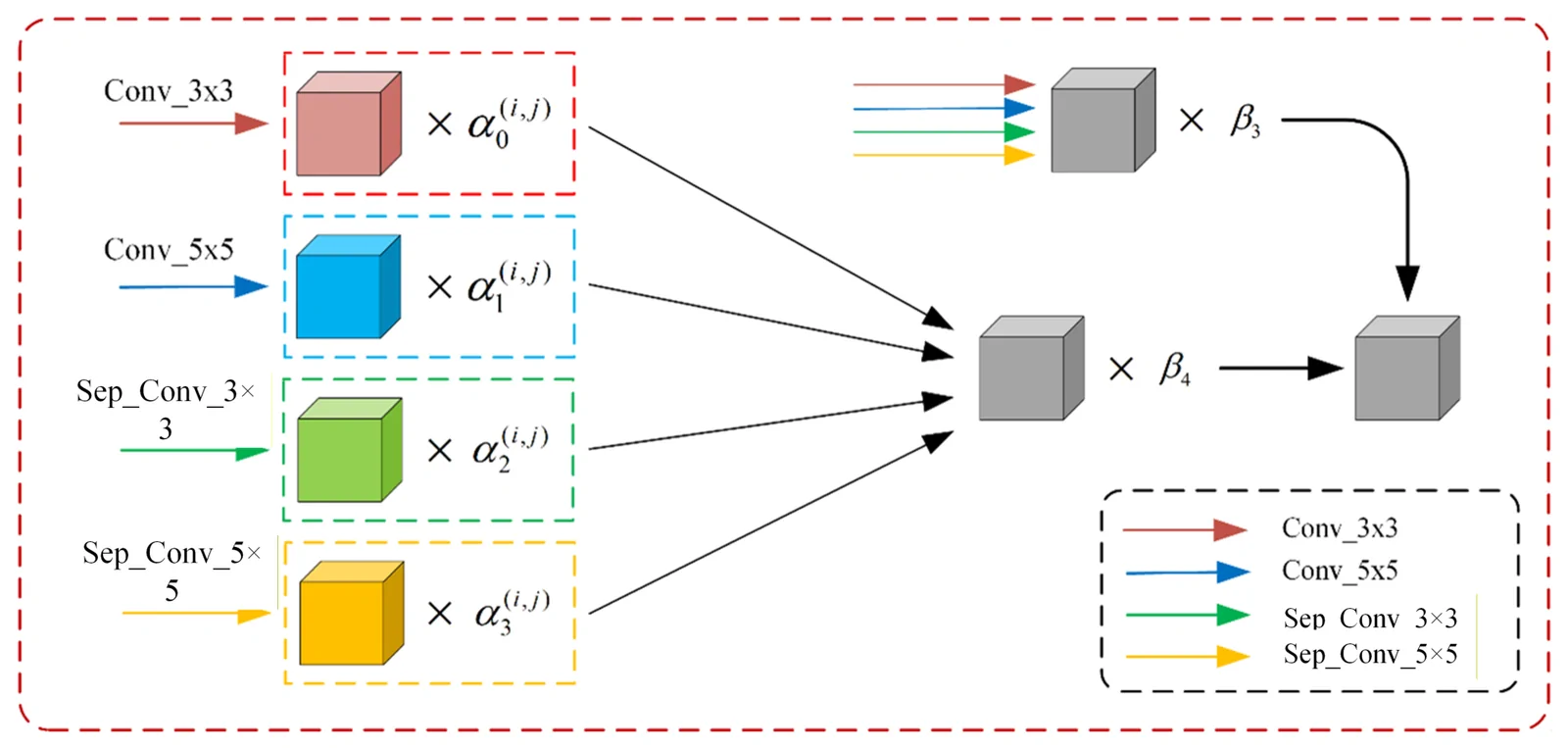
Dual optimization of operators and branches.

Note 1: The First layer and the Head are manually fixed; Cells 0–3 are searchable and share the same set of four candidate operators (Figure 4). A BN layer and a nonlinear activation follow every convolution (Section 3.2.3) and are omitted from the operator columns for brevity. Note 2: Channel counts separated by commas denote successive convolutions within the stage. Supernetwork widths are the pre-pruning values; final widths are obtained after BN-based channel pruning (Section 3.5). The seven output channels of the head correspond to four bounding-box offsets $\left(t_{x},t_{y},t_{w},t_{h}\right)$ and three class scores (background, lap-joint feature point, butt-joint feature point).

As detailed in Table 2, the network follows a pyramid structure in which each searchable cell halves the spatial resolution with a stride of two while the channel width of the supernetwork increases with depth. In contrast to architectures whose operator types are assigned manually per stage, all four searchable cells share the same candidate operator set—Conv 3 × 3, Conv 5 × 5, Sep_Conv 3 × 3 and Sep_Conv5 × 5 (Section 3.2.2)—so that the choice between standard and depthwise separable convolutions at each depth is left to the search rather than prescribed by design. Only the input layer and the prediction head are fixed, in order to keep input processing and output generation stable across candidate architectures. The operator and topology configuration actually selected by the search, together with the channel widths retained after pruning, is reported in the last two columns of Table 2 and analyzed in Section 4.3.

#### 3.2.2. Searchable Cells and Candidate Operators

**Figure 5:**
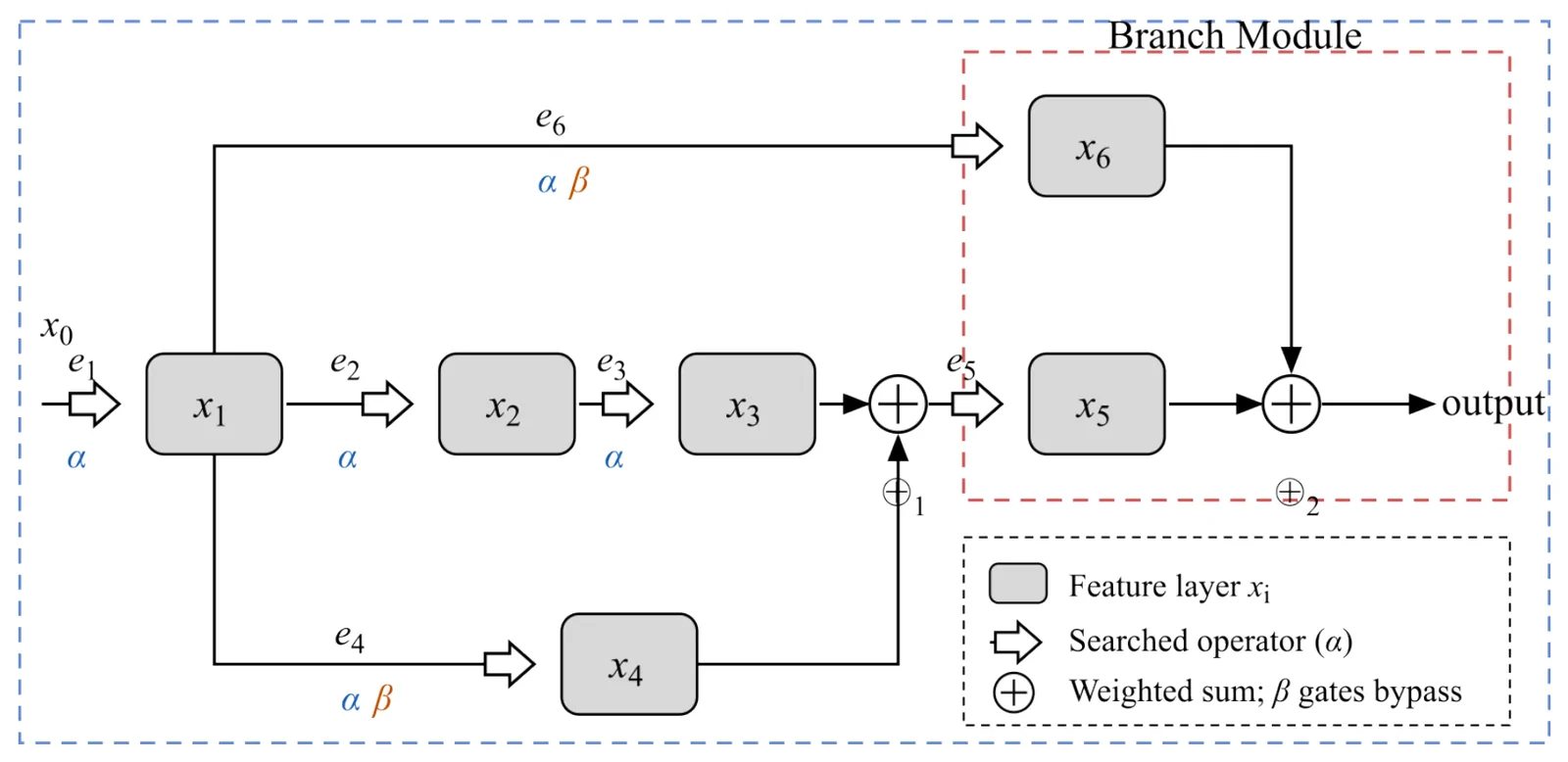
Internal architecture of a searchable cell. Solid boxes denote feature layers ($x_{i}$); labeled edges ($e_{j}$) carry either a searched operator (α) or a gated bypass connection (β); ⊕ denotes weighted summation.

To provide a flexible yet consistent topological framework, the four searchable Cells (Cell 0–Cell 3) share a unified internal structure. As depicted in Figure 5, following DARTS [28], each Cell is constructed as a directed acyclic graph (DAG) containing multiple feature processing paths. Whereas DARTS [10,28] search over the operators assigned to a fixed cell topology, the bypass edges e4 and e6 carry an additional gate β, so that the topology of the cell is itself subject to the search. The treatment of β as a variational parameter within the same posterior as α, and its coupling to the latency term, is developed in Section 3.3 and Section 3.4. Each cell contains seven feature layers connected by six directed edges: four sequential edges ($e_{1}$–$e_{3}$, $e_{5}$) that carry a searched operator, and two bypass edges ($e_{4}$, $e_{6}$) that additionally carry a gate β (Figure 5). All paths within a cell operate at the same resolution and channel width, so feature maps entering an aggregation node always match in dimension.

Each directed edge within this graph represents a candidate operation that transforms the input feature map. To balance feature extraction capability with computational efficiency, a search space consisting of four candidate operators is defined. The standard Conv 3×3 and Conv 5×5 are included to capture fine-grained local features and broader contextual information, respectively. Crucially, to achieve the lightweight design goals, Sep_Conv 3×3 and Sep_Conv 5×5 are introduced. These denote depthwise separable convolutions, which decompose a standard convolution into a depthwise convolution followed by a pointwise convolution. This decomposition significantly reduces the parameter count and floating-point operations (FLOPs) while maintaining representational power.

Feature map downsampling is implemented by setting the convolution stride to 2, effectively halving the spatial resolution at specific stages. To ensure seamless feature fusion within the multi-path structure, strict stride consistency is enforced across all branches; this prevents dimension mismatches when feature maps from different paths are aggregated. Since each bypass edge may be pruned when its β falls below the retention threshold, the searched cell may degenerate to a purely sequential structure; this is indeed what occurs in the final architecture (Section 4.3).

#### 3.2.3. Input Preprocessing and Normalization Design

The industrial camera of the laser vision sensor acquires images at a resolution of 1280 × 1024 pixels. Since the laser stripes and the seam feature point occupy only a small portion of the field of view, each raw image is cropped to a 192 × 192 region enclosing the laser stripe pattern before being fed to the network. This region is not a fixed window on the workpiece: in the closed-loop tracking system its position in each frame is set by the detection obtained in the preceding frame, as described in Section 4.4, so that the region travels with the torch and the joint is scanned continuously along its whole length as the robot traverses. Generating the region of interest from the detection in the preceding frame is established practice in seam tracking [16].

This preprocessing serves two purposes. First, it substantially lowers the computational load of both the architecture search and the final inference, which is essential for the targeted CPU-only deployment: the input tensor is reduced by a factor of approximately 35 relative to the full frame. Second, it standardizes the input distribution, so that the searched architecture is specialized to the appearance statistics of laser stripe patterns rather than to the surrounding background. What is specific to the present configuration is that the region is cropped rather than rescaled, so the imaging geometry is preserved and the scale factor of 0.0754 mm/pixel established in Section 3.1 remains valid for the network input; pixel-level positioning errors can therefore be converted directly into physical distances on the measurement plane.

Regarding normalization, a batch normalization (BN) layer [47] and a nonlinear activation are appended immediately after every convolution in both the fixed and the searchable modules, as summarized in Table 2. Besides accelerating convergence and stabilizing the bi-level optimization, this arrangement is a prerequisite for the channel selection mechanism: the per-channel scaling factors of these BN layers are reused as differentiable channel importance indicators, which allows redundant channels to be suppressed during the search itself rather than in a separate post-processing stage. This mechanism is detailed in Section 3.5.

#### 3.2.4. Feature Point Representation and Detection Head

The weld seam tracking task addressed in this work is formulated as the localization of a single feature point per frame. As defined in the annotation protocol (Section 4.1), this point is the corner point of the laser stripe formed at the weld seam, i.e., the intersection of the projected laser line and the seam profile. Since each laser-vision image contains exactly one such feature point, the detection problem is single-target by construction.

To make this point-localization task compatible with the SSD paradigm [35], the annotated feature point is represented as a bounding box centered at the point, and the network is trained to regress this box together with its class score. Accordingly, the prediction head is fixed and comprises two 3×3 convolutions: the first, with stride 2, maps the output of Cell 3 to 128 channels at a resolution of 7×7 and is followed by batch normalization and ReLU; the second, with stride 1, produces the seven prediction channels and is applied without normalization or activation, so that the regressed offsets and class logits remain unconstrained in sign. With a single anchor assigned per spatial location, the head yields at most 49 candidates per image, in contrast to the thousands of anchors typically employed in general-purpose SSD detectors. The head structure itself follows the SSD convention [35]; what is specific to the present formulation is its reduction to a single anchor per spatial location, which exploits the single-target nature of the task and contributes to the low inference cost reported in Section 4.2.

At inference, the candidate with the highest class confidence is retained, and the feature point is recovered as the center of the corresponding predicted box:

(1) $$\hat{u}=\hat{x}+\frac{\hat{w}}{2},\;\hat{v}=\hat{y}+\frac{\hat{h}}{2},$$

where $\left(\hat{x},\hat{y},\hat{w},\hat{h}\right)$ denotes the predicted box and $\left(\hat{u},\hat{v}\right)$ the recovered feature point. It should be emphasized that all accuracy metrics reported in this paper are therefore point-level rather than box-level: the positioning error of a frame is the Euclidean distance ${∥\left(\hat{u},\hat{v}\right)-\left(u,v\right)∥}_{2}$ between the recovered point and the annotated point (u,v), expressed in pixels, and no intersection over union (IoU)-based detection metric is used. Using the calibrated scale factor of 0.0754 mm/pixel (Section 3.1), these pixel-level errors are converted into physical errors on the measurement plane.

### 3.3. Bayesian Variational Architecture Search

To improve the exploration stability and global optimization capability of the architecture search process, this study adopts a probabilistic approach grounded in Bayesian learning rules. Conventional differentiable architecture search methods, such as DARTS, often suffer from instability and a tendency to collapse toward suboptimal solutions due to their reliance on deterministic gradient descent in the architecture parameter space. By treating architecture learning as a distribution learning problem, the proposed method naturally introduces stochasticity, which facilitates the exploration of superior model structures while reducing the risk of premature convergence [10].

The optimization process begins by defining architecture parameters for operators and network branches, denoted as $\alpha_{\theta }$ and $\beta_{\theta }$, respectively. In the Bayesian framework, the objective is to estimate the posterior distribution $p(\alpha_{\theta }|D)$ given the training dataset D. However, for deep neural networks with complex search spaces, this posterior distribution is computationally intractable due to the difficulty of calculating the normalization constant. Consequently, variational inference is employed to approximate the true posterior with a simple, tractable distribution $q(\alpha_{\theta })$.

To quantify the quality of this approximation, the Kullback–Leibler (KL) divergence [48] between the variational distribution $q(\alpha_{\theta })$ and the true posterior $p(\alpha_{\theta }|D)$ is utilized as the minimization objective. Minimizing this divergence is mathematically equivalent to maximizing the Evidence Lower Bound (ELBO) [49], a functional that serves as a proxy for the model evidence. The optimization objective function is formulated as follows:

(2) $$L\left(q\right)=-E_{q(\alpha_{\theta })}\left[\log p\left(D|\alpha_{\theta }\right)\right]+E_{q(\alpha_{\theta })}\left[\log\frac{q(\alpha_{\theta })}{p(\alpha_{\theta })}\right]$$

where the first term represents the expected log-likelihood of the data, which for the detection task corresponds to the negative detection loss $L_{train}$ (Section 3.2.4), and the second term acts as a regularizer penalizing deviation from the prior distribution $p(\alpha_{\theta })$. By maximizing the ELBO, the variational distribution $q(\alpha_{\theta })$ is iteratively refined to approach the true posterior.

For efficient optimization, the variational distribution $q(\alpha_{\theta })$ is modeled as a multivariate Gaussian distribution N(μ,diag(S)). In this probabilistic framework, the architecture parameters $\alpha_{\theta }$ (and similarly $\beta_{\theta }$) are treated as random variables sampled from this distribution. Since $\alpha_{\theta }$ and $\beta_{\theta }$ are Gaussian variates and may take negative values, they are not used directly as mixing weights. At each forward pass a sample is drawn and mapped to normalized weights

(3) $${\tilde{\alpha }}_{k}^{\left(i,j\right)}=\frac{\exp\;\left(\alpha_{\theta ,k}^{\left(i,j\right)}\right)}{\sum_{k^{\prime }=1}^{K}\exp\;\left(\alpha_{\theta ,k^{\prime }}^{\left(i,j\right)}\right)},\;{\tilde{\beta }}^{\left(i,j\right)}=\sigma \;\left(\beta_{\theta }^{\left(i,j\right)}\right),$$

where σ(·) denotes the logistic function. The normalized weights satisfy ${\tilde{\alpha }}_{k}^{\left(i,j\right)}\in \left(0,1\right)$ with $\sum_{k}{\tilde{\alpha }}_{k}^{\left(i,j\right)}=1$ and ${\tilde{\beta }}^{\left(i,j\right)}\in \left(0,1\right)$. These quantities, rather than the raw samples, enter the feature aggregation of Equation (6) and the latency penalty of Equation (7), so the logarithms in Equation (7) are well defined.

Consequently, the optimization objective shifts from directly tuning the discrete values of $\alpha_{\theta }$ to optimizing the continuous distribution parameters—specifically, the mean vector μ and the variance term *S*. Since standard gradient descent is often ill-suited for optimizing probability distributions directly, this study adopts natural-gradient updates which account for the geometry of the distribution space [50]. To ensure stable convergence, the parameters are updated using an Adam-like rule derived as follows [50,51]:

(4) $$\begin{array}{cc} u_{t+1}= & u_{t}+\lambda_{t}\left[\frac{1}{\sqrt{S_{t+1}}+\tilde{\lambda }I}\right]\left(g\left(\alpha_{\theta t}\right)+\tilde{\lambda }u_{t}\right)\\ \; & +\gamma_{t}\left[\frac{\sqrt{S_{t}}+\tilde{\lambda }I}{\sqrt{S_{t+1}}+\tilde{\lambda }I}\right]\left(u_{t}-u_{t-1}\right) \end{array}$$

(5) $$S_{t+1}=\left(1-\lambda_{t}\right)S_{t}+\lambda_{t}{\left[g(\alpha_{\theta t})\right]}^{2}$$

### 3.4. Dual-Selection Strategy with Latency Constraints

To effectively bridge the gap between high model accuracy and the strict real-time requirements of industrial welding, this study proposes a dual-selection optimization strategy. Conventional search strategies, such as those used in DARTS, often generate complex multi-branch topologies that hinder deployment on low-computing-power devices. Conversely, simplified architectures like FBNet may fail to fully exploit inter-layer feature information. To resolve this conflict, the proposed method jointly optimizes both the operation operators and the network branch structures.

As illustrated in Figure 5, the optimization process assigns a weight factor α to each candidate operator and a separate weight factor β to each branch structure.

The weight α determines the importance of a specific operator on a directed edge, while β evaluates the contribution of the entire branch to the network’s performance. During the forward pass, the input feature map is processed by all candidate operators. The final output of a feature layer, denoted as $F_{i}$, is computed as the weighted sum of these operator outputs, using the normalized operator weights $\tilde{\alpha }$ defined in Equation (3) The mathematical formulation for this weighted feature fusion is given by:

(6) $$F_{j}=\sum_{k=1}^{K}\left(F_{i}⊙K_{k}^{\left(i,j\right)}\right)\;\cdot \;{\tilde{\alpha }}_{k}^{\left(i,j\right)}$$

where $F_{i}$ is the feature map at node *i*, $K_{k}^{\left(i,j\right)}$ denotes the *k*-th candidate operation on edge (i,j), and ${\tilde{\alpha }}_{k}^{\left(i,j\right)}$ is the corresponding normalized operator weight from Equation (3). By optimizing these weights simultaneously, the network can dynamically select the most effective operator combinations while pruning redundant branches. Branches whose normalized gate ${\tilde{\beta }}^{\left(i,j\right)}$ falls below a predefined retention threshold are considered insignificant and are removed during the training process to simplify the final architecture.

Crucially, standard architecture search often prioritizes accuracy as the sole optimization objective, leading to computationally expensive models. To ensure the generated model meets the frame-rate demands of the welding system, inference latency is explicitly incorporated as a constraint. To avoid repetitive inference during the search process, a latency lookup table was constructed by pre-measuring the execution time of all candidate operators on the target Intel i7-3770 CPU. The total latency is subsequently estimated by summing the latencies of the selected operators and integrated into the loss function as a penalty term. It should be noted that this summation-based estimate neglects memory access, operator fusion, and framework scheduling overhead, and is therefore not intended as an accurate predictor of absolute end-to-end latency. Its role in the present framework is limited to providing a differentiable, relative ranking signal that steers the search toward low-latency operators and branches. The actual speed of the final architecture is verified by direct on-device measurement (Section 4.2). This mechanism compels the search algorithm to prioritize architectures that offer lower latency while maintaining acceptable accuracy. The resulting architecture loss function is defined as follows:

(7) $$\begin{array}{cc} L_{arch}=L_{train} & +\lambda_{\alpha }\sum_{\left(i,j\right)}\sum_{k}\log\;\left({\tilde{\alpha }}_{k}^{\left(i,j\right)}\;{\mathrm{Lat}}_{k}^{\left(i,j\right)}\right)\\ \; & +\lambda_{\beta }\sum_{\left(i,j\right)}\log\;\left({\tilde{\beta }}^{\left(i,j\right)}\;{\mathrm{Lat}}_{\beta }^{\left(i,j\right)}\right) \end{array}$$

Here, $L_{train}$ is the detection loss defined in Section 3.2.4, and ${\mathrm{Lat}}_{k}^{\left(i,j\right)}$, ${\mathrm{Lat}}_{\beta }^{\left(i,j\right)}$ denote the pre-measured inference costs of the candidate operator and of the bypass edge, respectively, taken from the latency lookup table. Since ${\tilde{\alpha }}_{k}^{\left(i,j\right)}$ and ${\tilde{\beta }}^{\left(i,j\right)}$ are strictly positive by construction (Equation (3)) and the tabulated latencies are positive, the logarithms are well defined.

### 3.5. Joint Channel Pruning Strategy

In addition to optimizing network topology, the number of channels in each feature layer significantly impacts both the model’s representation capability and its computational cost. However, searching for the optimal channel configuration is challenging; treating channel numbers as a discrete search variable results in an explosive search space that is computationally prohibitive to traverse. To address this, a structured channel pruning strategy is seamlessly integrated into the NAS process.

Instead of searching for exact channel counts, the method leverages the properties of the Batch Normalization (BN) layers appended after each convolution. The BN operation normalizes the input features using a scaling factor γ and a bias *b*, formulated as:

(8) $$y=\gamma \frac{x-\mu }{\sigma }+b$$

**Figure 6:**
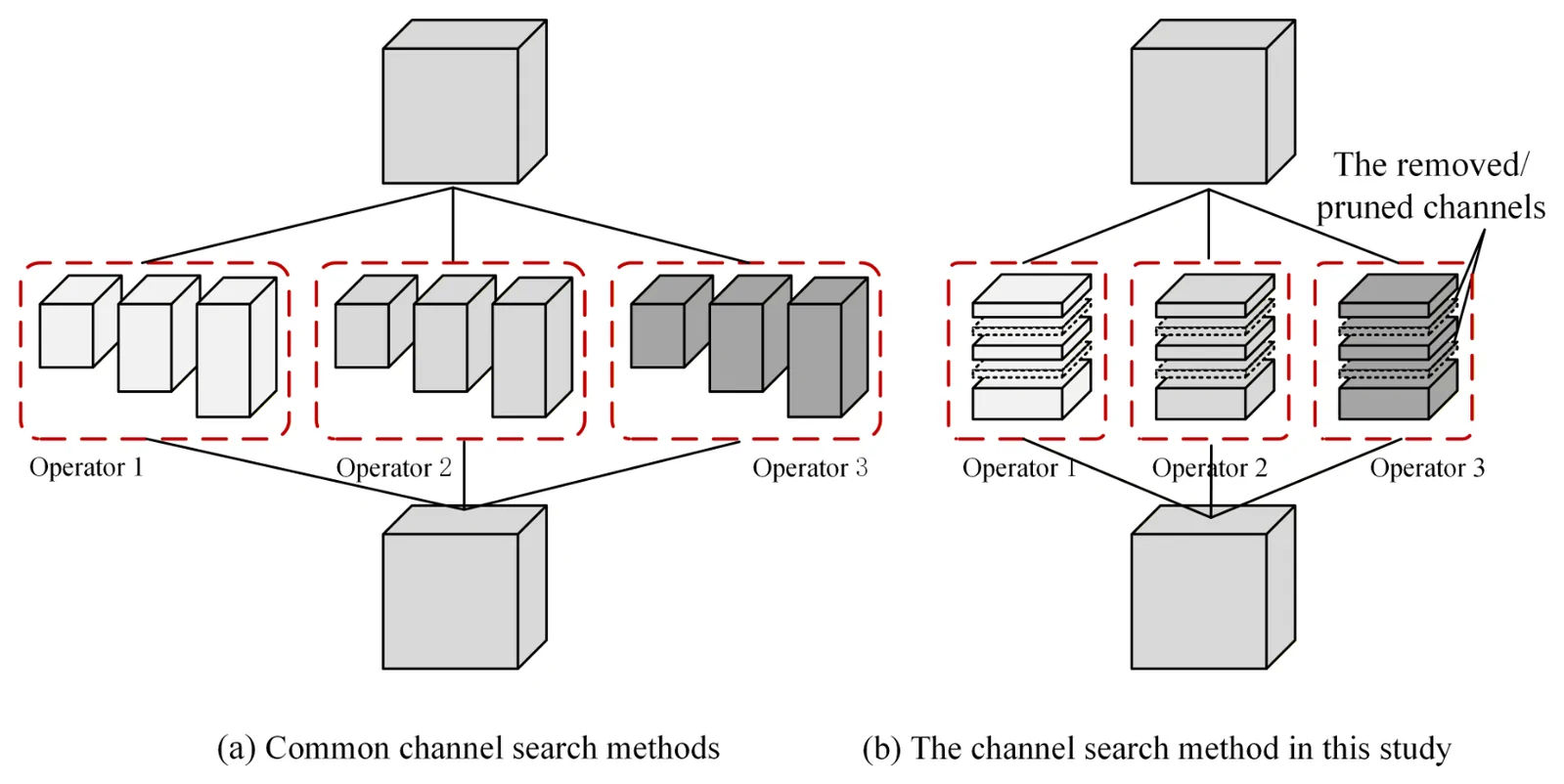
Comparison between the proposed channel pruning strategy and conventional channel search.

The scaling factor γ directly influences the activation magnitude of the corresponding channel. A value of γ close to zero implies that the channel contributes negligibly to the subsequent layers and can effectively be pruned. Figure 6 illustrates the distinction between this continuous pruning approach [34] and conventional discrete channel search methods.

To enforce sparsity and automate channel selection, $L_{1}$ regularization is applied to the scaling factors γ during supernetwork training. This regularization drives the scaling factors of unimportant channels toward zero, effectively “switching off” redundant features in a differentiable manner. Consequently, the total joint loss function for the supernetwork optimization combines the training loss, the latency constraints, and the channel sparsity penalty:

(9) $$L_{total}=L_{train}+\lambda_{Latency}\mathrm{Latency}\left(\tilde{\alpha },\tilde{\beta }\right)+\lambda_{c}\sum \left|\gamma \right|$$

where $\lambda_{c}$ is the weight coefficient for the $L_{1}$ regularization term. This joint optimization framework ensures that the final sampled subnetwork is not only topologically efficient and hardware-aware but also compact in terms of channel dimensions, thereby maximizing performance on resource-constrained industrial hardware.

### 3.6. Optimization Procedure and Model Extraction

**Figure 7:**
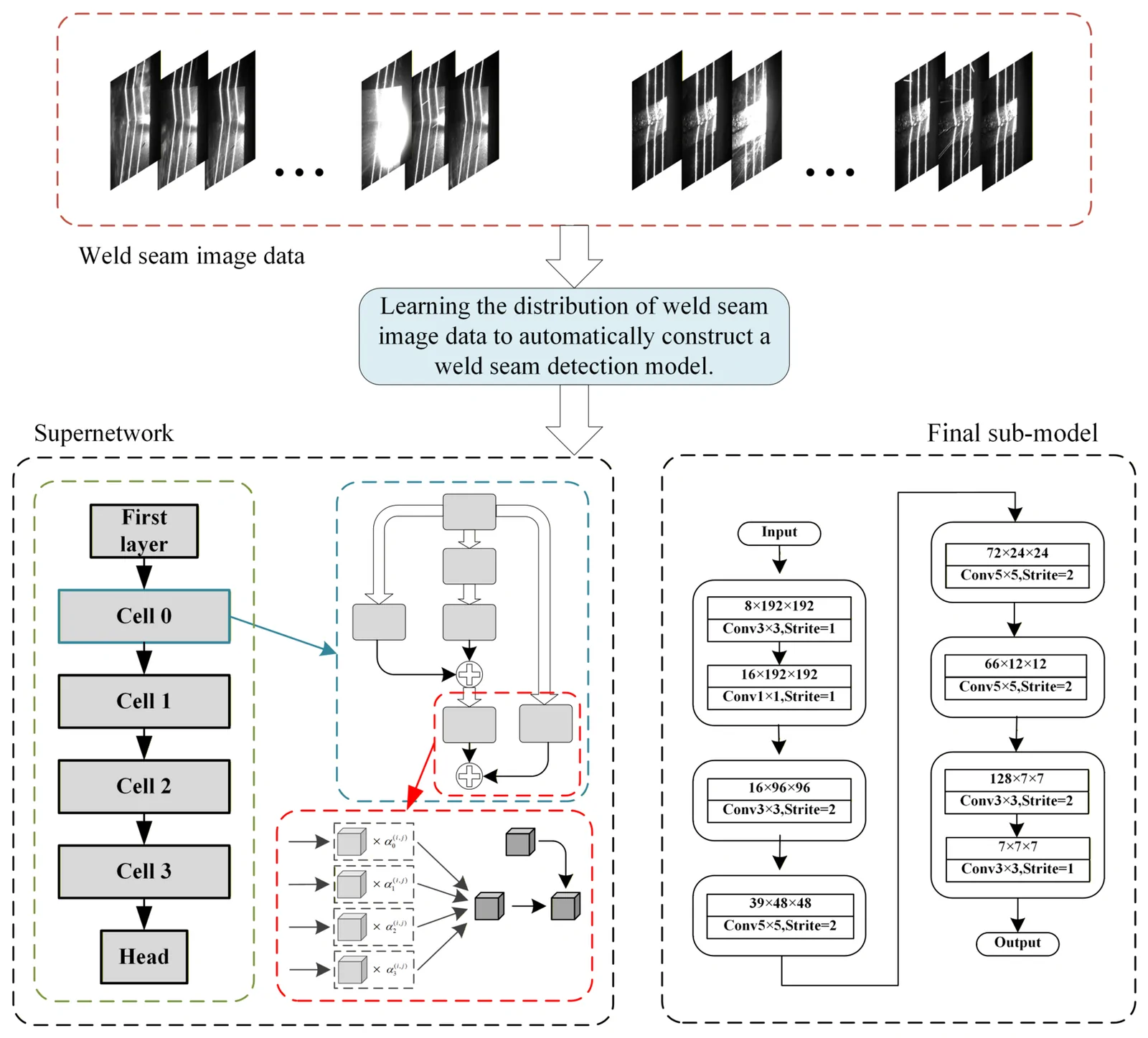
Optimization process of architecture parameters.

The training of the proposed supernetwork constitutes a bi-level optimization problem, requiring the simultaneous optimization of network weights θ and architecture parameters ($\alpha_{\theta },\beta_{\theta }$). To manage this complexity, an alternating optimization strategy is adopted, the complete workflow of which is illustrated in Figure 7. The supernetwork learns the distribution of weld seam image data through an iterative process. This process involves two alternating steps within each training epoch:

1. Weight Optimization: First, the architecture parameters $\alpha_{\theta }$ and $\beta_{\theta }$ are fixed. The network weights θ are updated using standard Stochastic Gradient Descent (SGD) [52] to minimize the detection loss on the training set.
2. Architecture Optimization: Subsequently, the network weights θ are fixed. The architecture parameters are optimized using the proposed Bayesian variational inference method. The gradients are estimated via Monte Carlo sampling, and the distribution parameters are updated using the natural gradient rule derived in Section 3.3.

The detailed training strategy is summarized in Table 3. Upon convergence, the final lightweight model is extracted from the supernetwork, as shown in the right panel of Figure 7. The optimal operators and branch connections are determined by sampling from the learned posterior distributions of $\alpha_{\theta }$ and $\beta_{\theta }$. Simultaneously, the channel pruning strategy (Section 3.5) takes effect: channels with near-zero scaling factors γ are removed. This results in a compact “Final sub-model” with specific, optimized channel configurations (e.g., varying from 72 to 128 channels across layers) that is significantly smaller than the original supernetwork. Finally, this subnetwork inherits the weights from the supernetwork and undergoes fine-tuning to maximize detection accuracy.

## 4. Experimental Results and Discussion

To rigorously validate the effectiveness of the proposed BayeNAS-Weld framework, comprehensive experiments were conducted. These included quantitative comparisons against state-of-the-art detection models on an offline dataset and practical tracking tests on a real-world robotic welding platform.

### 4.1. Experimental Setup and Implementation Details

**Figure 8:**
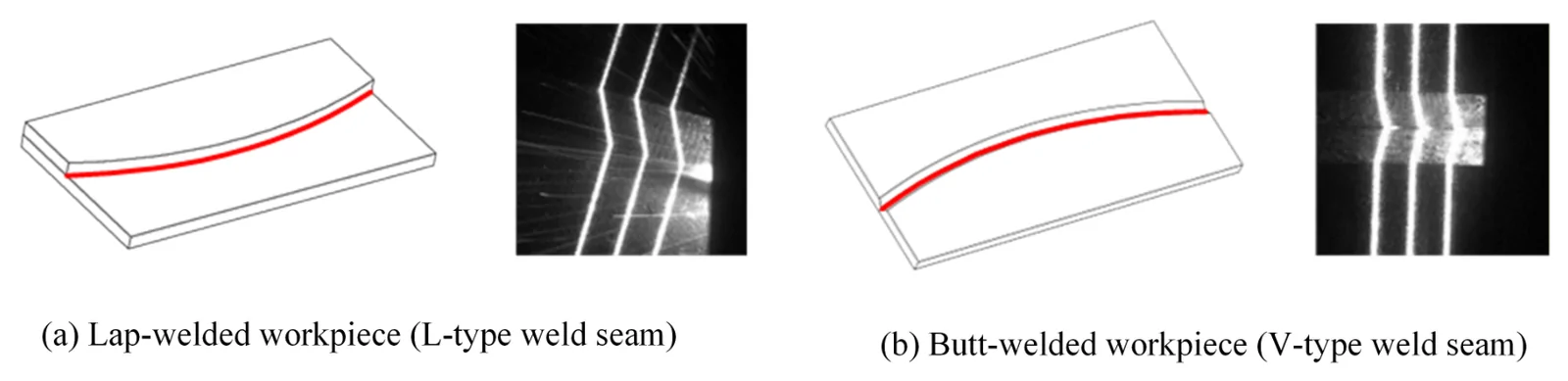
The two joint configurations used to construct the dataset, both prepared on flat 8 mm Q235 plates with straight seams.

To evaluate the proposed model under realistic industrial noise conditions, a large-scale weld seam dataset was constructed directly from a real-world welding environment characterized by severe noise interference, including strong arc light and metal spatter. The final dataset comprises a total of 19,814 images, partitioned into 14,848 training and 4966 test images in an approximate 3:1 ratio. The partition was made at frame level by random assignment, so that frames originating from the same welding sequence occur in both the training and the test partition. No separate validation partition was reserved: the architecture search and the choice of training schedule were carried out on the training partition, and the test partition was used only for the evaluation reported in Section 4.2. The implications of this arrangement are discussed in Section 4.6. The test set comprises 2455 lap-joint (L-type) and 2511 butt-joint (V-type) images, so the two joint types are represented in approximately equal proportion. Representative samples of these joint types and their corresponding trajectories are illustrated in Figure 8. The three-dimensional character of these joints resides in the groove and the step, which is what deforms the projected laser stripe and thereby creates the feature point to be located; the consequences of restricting the dataset to flat parent plates and straight seams are discussed in Section 4.6.

The experimental implementation was purposely divided into training and inference stages with distinct hardware configurations to align with actual industrial deployment constraints. Model training was conducted on a high-performance workstation equipped with an NVIDIA GeForce RTX 4080 GPU to ensure efficient supernetwork optimization. To rigorously validate the model’s feasibility on resource-constrained edge devices, however, all inference speed tests and real-world welding experiments were performed exclusively on a legacy Intel Core i7-3770 CPU (8 GB RAM) running Ubuntu 16.04, without any GPU acceleration. This setup simulates the limited computing power typically available in cost-effective industrial controllers. Inference of the proposed model was executed with a batch size of 1 on the 192 × 192 region of interest; the input resolutions used for the compared methods are given in Table 4. The reported FPS is defined as the average per-image network forward time measured over the complete test set of 4966 images, excluding image acquisition, preprocessing, and postprocessing, which are analyzed separately in Section 4.4.

The feature-point ground truth was annotated through a semi-automatic procedure: labels were created manually or propagated across consecutive frames by the efficient convolution operators (ECO) tracker [53], and all labels were subsequently verified and corrected by hand. Annotation was performed by a single annotator following a protocol that defines the feature point as the corner point of the laser stripe formed at the weld seam, i.e., the intersection of the projected laser line and the seam profile. Since the process relied on a single annotator, inter-annotator consistency was not assessed, and the unquantified annotation uncertainty remains a limitation of this study.

Regarding the training strategy, the supernetwork was optimized using a bi-level scheme over 100 epochs with a batch size of 32. The network weights were updated via Stochastic Gradient Descent (SGD) with an initial learning rate of 0.005, a momentum of 0.9, and a weight decay of 0.005. Simultaneously, the architecture parameters were optimized using the proposed Adam-based natural gradient method with a learning rate of 0.002. This decoupled optimization strategy ensures that the architecture parameters explore the search space effectively while the network weights converge to a stable solution.

### 4.2. Comparative Analysis on Offline Dataset

To evaluate the accuracy–efficiency trade-off of the proposed method, comparative experiments were conducted against a spectrum of representative object detection algorithms spanning three orders of magnitude in computational cost, from 0.089 to 102.8 GFLOPs. The selected baselines include the high-precision two-stage detector Faster R-CNN [54], as well as several representative lightweight one-stage detectors: FemtoDet [55], MobileNetV3-SSD [27,35], EfficientDet [56], and YOLOX-tiny [57]. The evaluation was performed comprehensively using four key metrics: Mean Euclidean Distance Error (Mean Error) to assess positioning accuracy, Standard Deviation (Std. Dev.) to evaluate stability, Frames Per Second (FPS) to measure inference speed on the target CPU, and model size metrics including Parameter count (Params) and Floating Point Operations (FLOPs).

The comparison was conducted at the system level: each detector was evaluated in the configuration in which it is normally deployed, that is, at its own native input resolution, while the proposed model operates on the 192 × 192 region of interest defined in Section 3.2.3. The input resolutions of all compared models are listed in Table 4. Each baseline was obtained from its publicly available implementation and trained on the training partition of Section 4.1. Since the baselines are general-purpose detectors that regress bounding boxes, their outputs were mapped to feature points in the same manner as those of the proposed model, by taking the center of the highest-scoring predicted box as in Equation (1); the accuracy figures of Table 4 are therefore point-level for every method, and no IoU-based detection metric is involved. Inference times were measured for all models on the same Intel Core i7-3770 CPU (Intel, Santa Clara, CA, USA) under the conditions stated in Section 4.1. The reported speed advantage should accordingly be read as the advantage of the complete sensing pipeline over these detectors as deployed, and not as a comparison of backbone efficiency at a fixed input size.

The quantitative evaluation results are summarized in Table 4. Regarding detection accuracy, the proposed BayeNAS-Weld achieves a mean positioning error of 1.739 pixels. This is slightly higher than YOLOX-tiny (1.496 pixels) and Faster R-CNN (1.642 pixels), while clearly lower than the other lightweight detectors, FemtoDet (3.552 pixels), EfficientDet (3.091 pixels), and MobileNetV3-SSD (1.806 pixels). It should be noted, however, that the two more accurate baselines require 6.44 GFLOPs and 102.8 GFLOPs respectively, whereas the proposed model requires only 0.128 GFLOPs. In other words, BayeNAS-Weld attains accuracy within 0.25 pixels of the best-performing detector at roughly 1/50 of its computational cost. In addition, the proposed model exhibits the lowest error standard deviation (1.064 pixels) among all compared methods, indicating a more concentrated error distribution on the test set and fewer large-deviation predictions than the other lightweight models. This metric reflects error dispersion on the evaluated images rather than robustness across independent welding conditions. Using the calibrated scale factor of 0.0754 mm/pixel (Section 3.1), the mean positioning error of 1.739 pixels corresponds to approximately 0.13 mm on the measurement plane.

In terms of computational efficiency, the proposed BayeNAS-Weld model demonstrates a significant advantage. To ensure that the measured latencies are comparable, all models were evaluated in a pure PyTorch (2.14.0) [58] environment without any hardware-specific acceleration library such as TensorRT or OpenVINO. Under this setting, the proposed model achieves an inference speed of 78.63 FPS, approximately 390 times faster than Faster R-CNN (0.20 FPS) and 12 times faster than YOLOX-tiny (6.34 FPS). This speed advantage is primarily explained by the drastic reduction in computational cost: the searched architecture requires only 0.128 GFLOPs, roughly 1/50 of YOLOX-tiny (6.44 GFLOPs), as a direct consequence of the latency-aware search objective. As shown in the last column of Table 4, the proposed model’s per-FLOP throughput (10.05 GFLOPS) is in fact lower than YOLOX-tiny’s (40.83 GFLOPS), which is expected for very small networks where framework overhead dominates; the overall speed advantage therefore stems from the reduction in total computation rather than higher hardware utilization. Moreover, FLOPs alone do not fully determine CPU latency—FemtoDet, despite its lower FLOPs (0.089 G), runs slower (16.66 FPS) than the proposed model, since per-layer framework overhead scales with network depth. The shallow, branch-pruned topology produced by the dual-selection strategy therefore contributes to the measured speed beyond the FLOPs reduction itself. All reported latencies are end-to-end measurements of the final networks; no hardware-level profiling (e.g., cache behavior analysis) was performed, and no such claim is made. For reference, a recently reported hand-designed lightweight detector for structured-light seam tracking attains mean absolute errors of 1.639–1.736 pixels at 29.32 ms per frame on a CPU [9]; a direct comparison is not possible because the datasets, noise conditions and annotation protocols differ, but the figures indicate that the searched architecture operates in the same accuracy regime at a lower inference cost. Chen et al. [16] address the same interference problem by geometric means—a dynamic region of interest propagated from the preceding frame combined with a slope-constrained RANSAC fit—rather than by a learned model, and report real-time seam extraction without a training stage. The distinction is one of operating regime. A geometric fit presupposes that enough inlier pixels survive to support a consistent line model; where that holds it is the preferable solution, requiring neither annotation nor training. The present work targets the regime in which that presupposition fails—where arc light saturates the stripe over part of its length, or spatter fragments it, so that no line model can be fitted to the surviving pixels and the feature point must be inferred from the appearance of the whole 192 × 192 neighbourhood, including regions in which the stripe is absent. The cost of that capability is the requirement for annotated data, and no claim of superiority is made: the datasets, noise conditions and evaluation metrics of the two studies differ, and the method of Chen et al. [16] was not evaluated on the present data.

The frame rate that a detector must reach in this application is set by the sensing configuration rather than by the model. The camera acquires at 60 fps (Table 1), so that at the traverse speed of 15 mm/s the joint is profiled every 0.25 mm. A detector slower than 60 FPS therefore becomes the bottleneck of the tracking loop, and the correction transferred to the torch is computed from a measurement that is already stale. None of the five general-purpose baselines meets this requirement on the target controller—the fastest, MobileNetV3-SSD, reaches 24.25 FPS—whereas the searched architecture reaches 78.63 FPS and leaves the acquisition rate as the limiting factor.

### 4.3. Analysis of Optimization Components

The following analysis examines the architecture that the constrained search produced and the computational consequences of the selections it made; it is not a substitute for a component-wise ablation, which is identified as a limitation in Section 4.6. The favorable speed–accuracy–size trade-off of BayeNAS-Weld can be traced to the two mechanisms introduced in Section 3: the latency-aware dual-selection strategy and the joint channel pruning. The following examines the architecture actually produced by the search, shown in the right panel of Figure 7.

The latency term in the architecture loss shaped the final topology in three observable ways. First, the search retained standard convolutions in all four cells rather than defaulting to depthwise separable operators, and instead controlled the computational cost through kernel size and resolution: a 3 × 3 kernel was selected for Cell 0, which operates at the highest resolution (96 × 96), whereas 5 × 5 kernels were selected for Cells 1–3, where the feature maps have already been reduced to 48 × 48 and below. Since the cost of a convolution scales with $H\times W\times k^{2}$, this allocation spends receptive field where it is inexpensive and economizes where it is not; had a 5 × 5 kernel been selected for Cell 0 instead, that single layer alone would have required approximately 59 M multiply–accumulate operations rather than 21 M. As a result, the per-layer cost of the four cells remains within a narrow range of roughly 17–40 M multiply–accumulate operations, indicating a balanced rather than a bottlenecked design. Second, the branch selection reduced the multi-path cell structure of the supernetwork to a single linear chain, eliminating the parallel paths and aggregation nodes that would otherwise incur additional memory traffic on a CPU. Third, the selected operator and branch configuration yields a total of 0.128 GFLOPs, roughly one fiftieth of YOLOX-tiny (Table 4). These observations describe the architecture that the constrained search produced; isolating the individual contribution of the latency term would require a controlled comparison against an unconstrained search, which is identified as a limitation in Section 4.6.

Furthermore, the joint channel pruning strategy significantly compressed the model size while maintaining its representational capacity. Conventional channel search methods typically treat channel counts as discrete variables, resulting in a combinatorial explosion of the search space. In contrast, the proposed method leverages $L_{1}$ regularization on the Batch Normalization scaling factors to implement a continuous, differentiable pruning process. As illustrated in Figure 6, this approach allows the network to automatically identify and suppress redundant feature channels during training. The retained widths differ markedly across depths —16 of 32 channels in Cell 0, 39 of 64 in Cell 1, 72 of 128 in Cell 2 and 66 of 128 in Cell 3 (Table 2)—confirming that a uniform channel configuration would have been suboptimal for this task.

The effectiveness of this strategy is evidenced by the final model statistics: the proposed model contains only 0.292 million parameters, a fraction of the size of compact models like MobileNetV3-SSD (2.657 M). This compactness indicates that the pruning mechanism successfully removed unnecessary redundancy, leaving a streamlined architecture that is highly optimized for the specific visual patterns of weld seam images. Consequently, the combination of topological simplification via latency constraints and dimensional reduction via channel pruning together account for the observed 78.63 FPS. A component-wise ablation quantifying their separate contributions is left for future work.

### 4.4. Real-World Robotic Welding Experiments

To verify the practical applicability and robustness of the proposed algorithm under actual production conditions, rigorous welding experiments were conducted using a Gas Metal Arc Welding (GMAW) system. The experiments utilized Q235 steel as the base material, with a shielding gas mixture of 20% CO_2_ and 80% Argon. The specific process parameters are detailed in Table 5. See Welding errors in Table 6.

**Figure 9:**
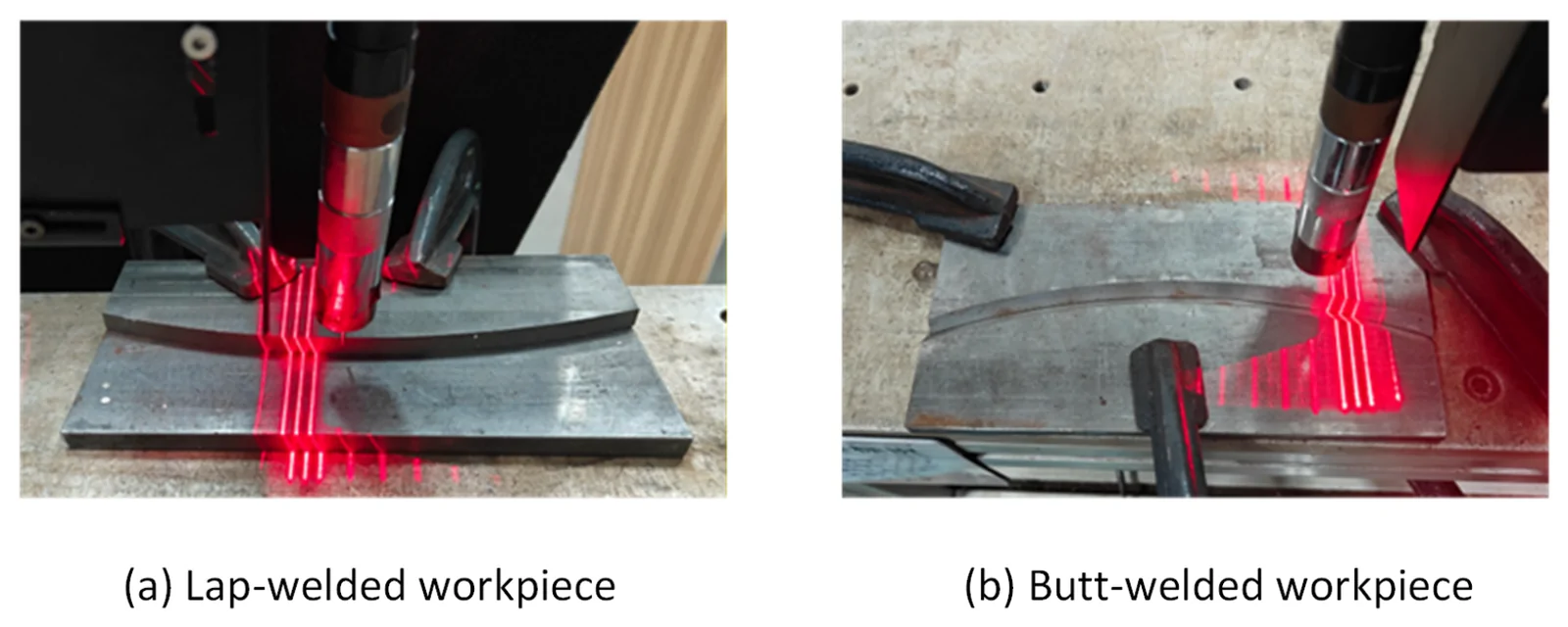
Clamping setup for welding experiments.

The physical experimental setup is illustrated in Figure 9. As shown, two typical industrial joint configurations were prepared: a lap-welded workpiece (L-type) and a butt-welded workpiece (V-type). The clamping setup ensures the stability of the workpiece during the welding process, serving as the basis for evaluating the tracker’s precision.

**Figure 10:**
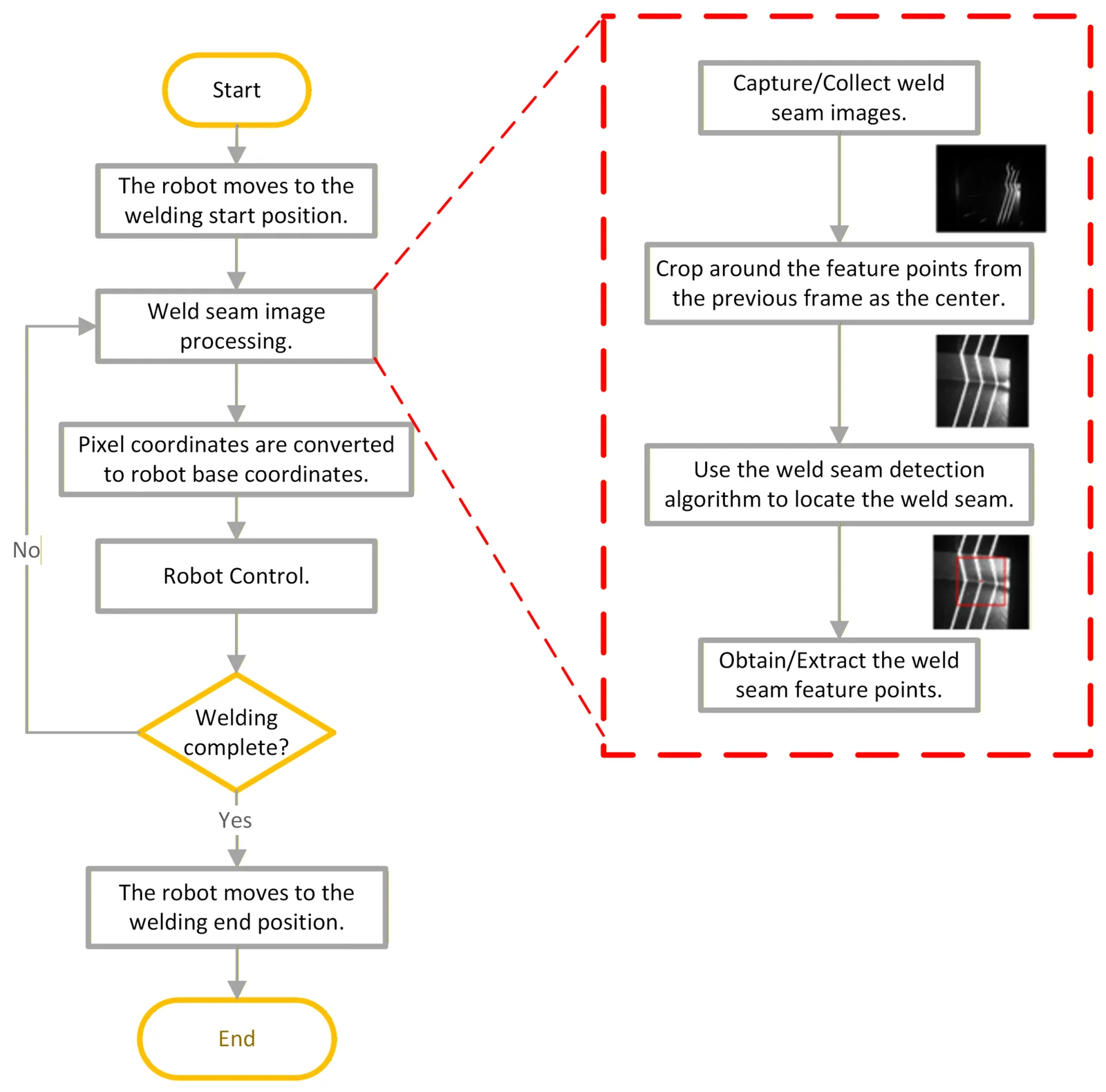
Overall workflow of weld seam tracking.

The complete automated tracking system operates according to the workflow depicted in Figure 10. The system executes a closed-loop process involving image acquisition, ROI cropping based on the previous frame, real-time seam detection using the proposed BayeNAS-Weld, coordinate transformation, and robot motion control. To facilitate quantitative error analysis, a reference trajectory was established prior to welding by manually teaching the robot along the seam with the welding power source deactivated. Each waypoint was verified by bringing the welding torch tip into physical contact with the reference point and recording the corresponding torch coordinates from the robot controller; a smooth curve was then fitted to the recorded waypoints. The robot’s specified position repeatability is ±0.08 mm. It should be noted that the reported tracking error therefore reflects the combined effect of detection error, calibration residuals, manual teaching accuracy, and robot positioning error, rather than the detector error alone.

**Figure 11:**
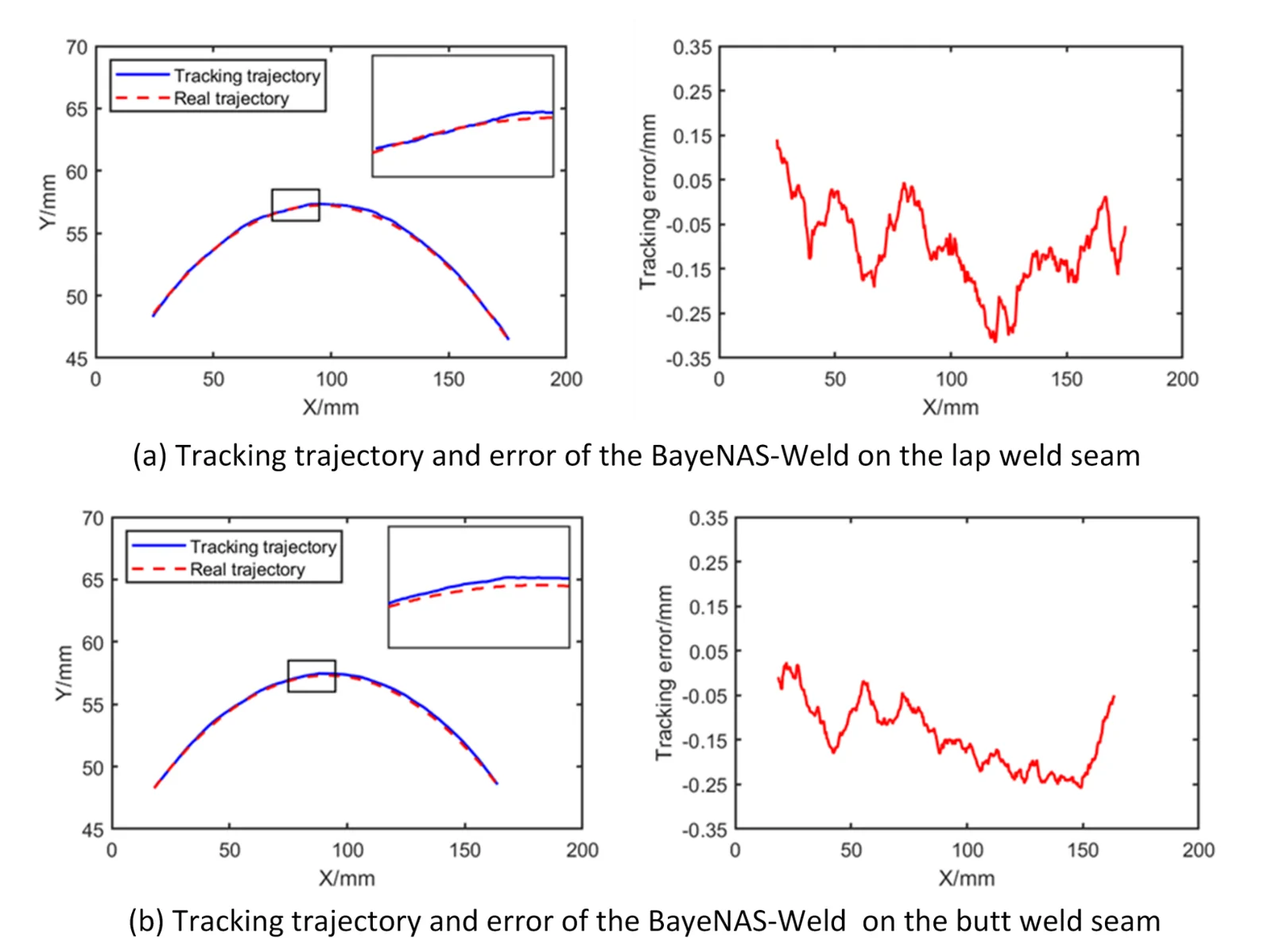
Closed-loop tracking result for (**a**) the lap joint and (**b**) the butt joint. Left: tracked trajectory versus the manually taught reference trajectory, with an enlarged view of the boxed region. Right: signed tracking error along the seam.

Figure 11 shows the tracked and reference trajectories together with the signed tracking error for one lap-joint and one butt-joint weld. The tracked seams span approximately 150 mm and 145 mm along the X axis of the robot base frame, corresponding to about 10 s of welding at the traverse speed of 15 mm/s given in Table 5. The mean absolute error was 0.1108 mm for the lap joint and 0.1442 mm for the butt joint. The signed error, however, is predominantly negative, so the deviation is better described as a systematic offset than as zero-mean noise. This is most evident for the butt joint, where the maximum positive excursion over the entire trajectory was only +0.024 mm while the maximum negative excursion reached −0.2587 mm.

Two features of the error curves deserve comment. First, the error does not converge to zero along the trajectory: in both cases it drifts negatively over the first two thirds of the seam, reaching −0.3158 mm for the lap joint and −0.2587 mm for the butt joint, and then partially recovers toward the end. A negative bias of this magnitude is of the same order as the hand-eye calibration residual reported in Section 3.1 (below 0.5 mm), and may additionally reflect accumulated displacement of the previous-frame ROI or a constant offset in the manually taught reference trajectory; the present data do not permit these contributions to be separated. Second, the error remains bounded within ±0.32 mm throughout both welds despite intermittent arc light and metal spatter. A bounded error on two individual trajectories constitutes a proof-of-concept demonstration rather than evidence of general robustness: establishing invariance to arc light and spatter would require repeated welds over multiple workpieces and a comparison against alternative detectors within the same closed loop, which is identified as a limitation in Section 4.6.

**Figure 12:**
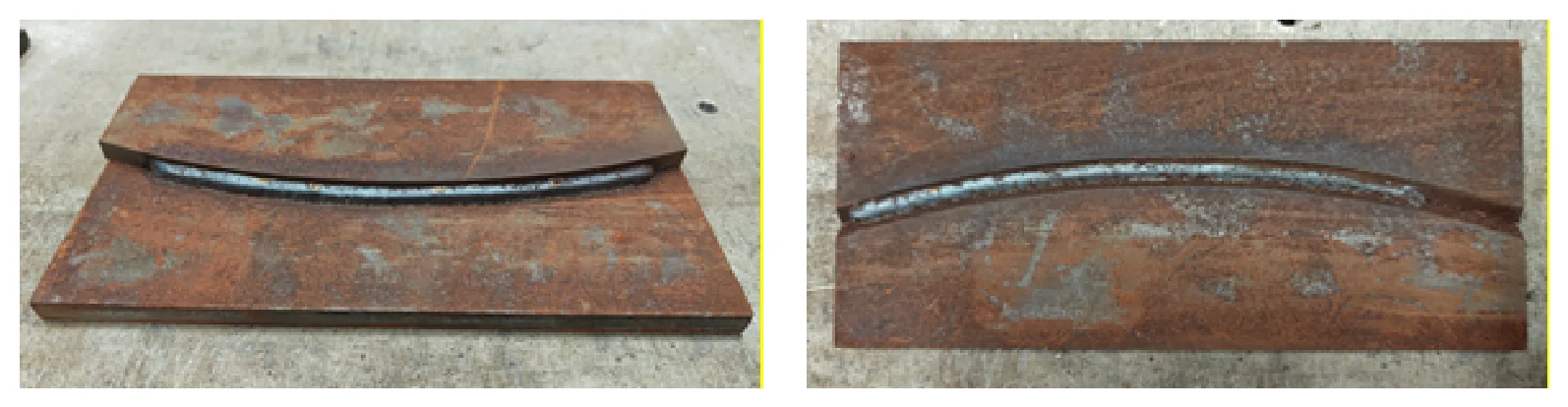
Final welding results.

Figure 12 shows the appearance of the completed welds. The beads are continuous and visually uniform along both seams, and no surface irregularities such as undercut, visible discontinuity or bead wandering were observed. This surface examination serves only to confirm that closed-loop tracking produced a continuous bead; it is a visual assessment of the weld surface only: no cross-sectional metallography, penetration measurement, or quantitative bead geometry measurement was carried out, and therefore no claim is made regarding internal weld quality. Together with the tracking results of Figure 11, these observations indicate that the detector is able to support closed-loop welding under the tested conditions.

### 4.5. Error Budget and Positioning Accuracy

The influence of the calibration residual is best seen by asking where the calibration enters. The detector operates entirely in the image plane: the positioning errors of Section 4.2, and with them the architecture search, the model selection and the comparison of Table 4, are pixel-domain quantities measured against pixel-domain annotations, and are therefore independent of the calibration. Calibration enters at two points only—the scale factor of 0.0754 mm/pixel, which expresses those pixel errors in millimeters, and the sensor-to-base transform, which is used only in the closed-loop experiments of Section 4.4.

Writing the feature point in the robot base frame as $p_{b}=Rp_{s}+t$, where $p_{s}$ is the point measured in the sensor frame and (R,t) the hand–eye transform, a perturbation gives $\delta p_{b}=R\delta p_{s}+\delta R\;p_{s}+\delta t$. The first term is the detection error transported into the base frame; rotation preserves its magnitude, so it contributes 0.131 mm for the mean pixel error of 1.739 px and 0.080 mm for its standard deviation of 1.064 px, and it varies from frame to frame. The remaining two terms constitute the calibration error. For a fixed tool orientation $p_{s}$ varies only within the 14.5 × 14.5 mm field of view, so that $\delta R\;p_{s}+\delta t$ is very nearly constant along the trajectory: a residual calibration error appears as a rigid offset of the tracked path rather than as additional noise. Its magnitude is bounded by the residual reported in Section 3.1, below 0.5 mm; interpreted as dominated by rotation at the working distance of 120 mm, this corresponds to an angular error below approximately 0.24°. An error ε in the scale factor contributes at most ε times the half-field of view, that is 0.07 mm for a 1% error, and perturbs the reported mean error of 0.131 mm by 1% of its value; it is negligible by comparison.

Table 7 sets these contributions against the accuracy of the robot itself and against the measured outcome.

This decomposition is consistent with the closed-loop measurements. The detection error and the robot repeatability are of the same order as the measured closed-loop error, whereas the calibration residual is larger than it; the closed-loop performance is therefore not limited by the robot, whose specified repeatability of ±0.08 mm is comparable to the standard deviation of the detection error itself, but by the systematic terms in the chain. The signed errors of Figure 11 are accordingly predominantly of one sign—for the butt joint the largest positive excursion over the entire trajectory is +0.024 mm while the largest negative excursion reaches −0.2587 mm—which is the signature of a rigid offset rather than of zero-mean noise, and the magnitude of that offset, 0.26 to 0.32 mm, is of the same order as, and below, the calibration residual. Since the random contribution of the detector, at 0.080 mm, is between three and four times smaller than the observed offset, that offset cannot be attributed principally to the detector.

A further property of the system, previously left implicit, is that the tracking error does not accumulate. Each frame yields an absolute measurement of the feature point in the sensor frame, which is mapped into the base frame through the fixed hand–eye transform; the detection obtained in the preceding frame determines only the position at which the current frame is cropped (Section 3.2.3) and does not enter the coordinate computation. Detection errors are therefore independent from frame to frame and do not integrate along the seam, so the positioning accuracy required of the robot is not compromised by the length of the weld. The bounded behavior of the error over the 150 mm and 145 mm trajectories of Figure 11 is consistent with this.

A bias of the kind identified above is in principle removable by recalibration or by a constant offset correction, whereas the random component is not. These statistics are, however, obtained from a single weld per joint type, and the present data do not allow the systematic term to be separated among calibration, manual teaching and control; both points are stated in Section 4.6.

### 4.6. Limitations

The scope of the reported results is delimited by the following limitations.

**Search and evaluation.** The reported architecture is the outcome of a single search run. Since the architecture posterior is sampled stochastically, neither the stability of the selected topology across random seeds nor confidence intervals for the resulting accuracy, speed and model size could be established. A component-wise ablation—deterministic versus variational search, operator-only versus joint operator-and-branch selection, and search with and without the latency and channel-pruning terms—was likewise not performed, so the individual contribution of each mechanism cannot be quantified from the present data. The per-operator latency lookup table was used only as a relative ranking signal during the search and was not validated against measured latencies of intermediate candidate architectures. The baselines of Table 4 were selected to span three orders of magnitude of computational cost rather than to represent the current accuracy frontier, and a comparison against the most recent general-purpose detectors, such as the YOLOv8 and YOLO11 families, under a common training and measurement protocol was not performed.

**Data.** The frame-level partition described in Section 4.1 means that the offline accuracy reported in Section 4.2 may overestimate generalization to unseen workpieces, lighting conditions or acquisition sessions; no separate validation partition was reserved, and the ground truth was produced by a single annotator, so the annotation uncertainty remains unquantified.

**Closed-loop validation.** The closed-loop experiments comprise one weld per joint type on a single workpiece pair. Consequently, failure rates, across-repetition variability and a comparison against alternative detectors within the same control loop are not available: as noted in Section 4.5, the systematic component of the error can be bounded but not attributed to a specific source among calibration, manual teaching and control. Weld quality was assessed from surface appearance only; no cross-sectional examination or penetration measurement was carried out.

**Workpiece geometry.** The dataset and the closed-loop experiments are confined to lap and butt joints prepared on flat 8 mm Q235 plates with straight seams. Three categories of more complex structure were therefore not examined. Additional joint types, such as fillet, T and multi-pass joints, alter the local shape of the stripe at the feature point; within the present framework, accommodating them requires additional training data and an increase in the number of class scores produced by the head, from the three used here to one per joint type, but leaves the searched backbone unchanged, and single frameworks covering butt, V-groove and multi-pass seams have been demonstrated in the same sensing modality [19]. Curved parent surfaces are the more demanding case: the profile sampled from a curved surface varies with the viewing direction, so that the appearance statistics to which the searched architecture is specialized (Section 3.2.3) no longer hold; compensating for this projection distortion is itself an active subject [59]. Curved and spatial seam trajectories, finally, affect the tracking loop rather than the detector, since the forerun distance of Section 3.1 couples to the local curvature of the seam and the region of interest must continue to contain the stripe, a requirement that is addressed elsewhere by global path planning [17]. It is worth noting that adapting to a new joint geometry or a new sensing configuration amounts, within the present framework, to repeating the search on the corresponding data rather than to redesigning the network by hand, which is the situation the method was constructed to address.

## 5. Conclusions

This study addressed the trade-off between positioning accuracy and real-time inference in laser-vision-based robotic welding. This paper presented BayeNAS-Weld, a lightweight weld seam feature point detector constructed by Bayesian variational neural architecture search, in which a latency-aware dual-selection mechanism searches jointly over candidate operators and topology branches and BN-based channel pruning is applied inside the search loop rather than as a post-processing step.

On an offline test set the searched model attains a mean positioning error of 1.739 pixels, corresponding to approximately 0.13 mm on the measurement plane. This is within 0.25 pixels of the most accurate baseline while requiring 0.29 M parameters and 0.128 GFLOPs, roughly one seventeenth of the parameters and one fiftieth of the computation of YOLOX-tiny, and it runs at 78.63 FPS on a legacy Intel Core i7-3770 CPU without GPU acceleration. In closed-loop GMAW experiments the mean absolute tracking error was 0.1108 mm for a lap joint and 0.1442 mm for a butt joint, with deviations bounded within 0.32 mm. The contribution of this work therefore lies in the accuracy–latency–size trade-off achieved by the integrated search framework rather than in absolute detection accuracy.

Future work will address the limitations identified in Section 4.6, in particular repeated searches for statistical reliability and a component-wise ablation, and will extend the search space beyond convolutional operators toward attention-based blocks and multi-task formulations that combine seam tracking with defect detection.

## Figures and Tables

**Table 1:** Components and parameters of the laser vision sensor.

No.	Component	Model/Material	Key Parameters
1	Triple-line laser generator	NL-660-100-03L-30	660 nm, 100 mW, fan angle 30°, three parallel lines
2	Industrial camera	Basler acA1280-60gm	e2v EV76C560 CMOS, 1280 × 1024, 60 fps
3	Camera lens	Computar M0814-MP2	*f* = 8 mm, F1.4–F16, 2/3″ format
4	Optical filter	BNP660	Bandpass, center 660 nm, bandwidth 10 nm
5	Protective glass	PCTSP4H-70-40-3-F60	Polycarbonate, total transmittance 91%
6	Protective housing	Aluminum alloy	—

**Table 3:** Model training algorithm.

Step	Description
Initialization	Initialize architecture parameters $\alpha_{\theta },\beta_{\theta }$ and network weights θ.
Optimization Loop	1. Fix $\alpha_{\theta },\beta_{\theta }$; optimize θ and apply $L_{1}$ regularization to BN scaling factor γ.
	2. Fix θ; optimize $\alpha_{\theta },\beta_{\theta }$ using the proposed Bayesian method.
Model Extraction	Derive the optimal subnetwork based on the converged distributions of $\alpha_{\theta },\beta_{\theta }$ and γ.

**Table 4:** Comparative evaluation on the offline weld seam test set.

Method	Input Resolution	Mean Error/Pixel	Std. Dev./Pixel	FPS (CPU)	Params/M	FLOPs/G	Effective Throughput/GFLOPS
Faster-RCNN	800×1333	1.642	1.551	0.20	39.43	102.8	20.56
FemtoDet	192×192	3.552	10.08	16.66	0.0676	0.08937	1.49
Mobilev3-SSD	192×192	1.806	1.503	24.25	2.657	0.2477	6.01
EfficientDet	512×512	3.091	1.419	1.95	3.828	2.120	4.13
YOLOX-tiny	416×416	1.496	2.763	6.34	5.030	6.440	40.83
**BayeNAS-Weld (ours)**	192×192	**1.739**	**1.064**	**78.63**	**0.2919**	**0.1278**	**10.05**

Note: Each model was evaluated at the input resolution at which it is normally deployed. Effective throughput = FPS × FLOPs, reflecting the realized hardware utilization of each model on the target CPU.

**Table 5:** Experimental platform and welding parameters.

Item	Model/Value
*Robotic platform*	
Welding robot	Yaskawa MOTOMAN-MA1440-C
Robot position repeatability	±0.08 mm ^1^
Robot controller	Yaskawa DX200
Welding power source	Yaskawa MOTOWELD-RD350
*Welding parameters*	
Welding process	GMAW
Power-source open-circuit voltage	100 V ^2^
Welding current	110 A
Welding speed	15 mm/s
Wire diameter	1.2 mm
Shielding gas	20% CO_2_ + 80% Ar
Workpiece material	Q235
Workpiece size	200 mm × 100 mm × 8 mm

^1^ Manufacturer specification. Its relation to the measured closed-loop tracking error is analyzed in Section 4.5. ^2^ The 100 V value is the open-circuit voltage setting of the power source, not the arc voltage during welding. The arc voltage was not logged during the experiments; for GMAW at the current level used here it is typically of the order of 20 V.

**Table 6:** Welding errors.

Model	Weld Seam Type	Mean Abs. Error (/mm)	Upper Error Bound (/mm)	Lower Error Bound (/mm)
BayeNAS-Weld	Lap joint	0.1108	0.1403	−0.3158
	Butt joint	0.1442	0.0241	−0.2587

Note: Errors are signed lateral deviations of the tracked feature point from the reference trajectory. The upper and lower error bounds are the maximum positive and maximum negative signed errors along the trajectory. Statistics are computed over a single weld per joint type.

**Table 7:** Contributions to the closed-loop tracking error, and the measured outcome.

Contribution	Magnitude	Character	Source
Feature-point detection (mean)	1.739 px = 0.131 mm	Random	Table 4
Feature-point detection (std. dev.)	1.064 px = 0.080 mm	Random	Table 4
Hand–eye calibration residual	<0.5 mm	Systematic	Section 3.1
Robot position repeatability	±0.08 mm	Random	Manufacturer
Manually taught reference trajectory	Not quantified	Systematic	Section 4.4
Measured closed-loop mean absolute error	0.1108/0.1442 mm	—	Table 6
Measured closed-loop extreme deviations	+0.024/−0.3158 mm	—	Table 6

Values separated by a slash refer to the lap joint and the butt joint, respectively.

## Data Availability

The data are available from the corresponding author upon reasonable request.

## Abbreviations

The following abbreviations are used in this manuscript:

BayeNAS	Bayesian Variational Neural Architecture Search
BN	Batch Normalization
cGAN	Conditional Generative Adversarial Network
CPU	Central Processing Unit
DAG	Directed Acyclic Graph
DARTS	Differentiable Architecture Search
ECO	Efficient Convolution Operators
ELBO	Evidence Lower Bound
FLOPs	Floating Point Operations
FPS	Frames Per Second
GMAW	Gas Metal Arc Welding
GPU	Graphics Processing Unit
IoU	Intersection over Union
IPCE	Internal Propulsion Center Extraction
KL	Kullback–Leibler
LoG	Laplacian of Gaussian
LSEGN	Laser Stripe Edge Guidance Network
LSFP	Laser Stripe Feature Point
NAS	Neural Architecture Search
NMS	Non-Maximum Suppression
NURBS	Non-Uniform Rational B-Splines
R-CNN	Region-based Convolutional Neural Network
ReLU	Rectified Linear Unit
ROI	Region of Interest
SDP	Semidefinite Programming
SGD	Stochastic Gradient Descent
SOLO	Segmenting Objects by Locations
SSD	Single Shot MultiBox Detector
VGG	Visual Geometry Group
